# Impact of government interventions on the stock market during COVID-19: a case study in Indonesia

**DOI:** 10.1007/s43546-022-00312-4

**Published:** 2022-08-17

**Authors:** Josua Sinaga, Ting Wu, Yu-wang Chen

**Affiliations:** Alliance Manchester Business School, The University of Manchester, Manchester, M15 6PB UK

**Keywords:** COVID-19, Financial markets, Emerging country, Government interventions, Stock return

## Abstract

This paper aims to examine the short-term impact of government interventions on 11 industrial sectors in the Indonesian Stock Exchange (IDX) during the COVID-19 pandemic. Whereas earlier studies have widely investigated the impact of government interventions on the financial markets during the pandemic, there is lack of research on analysing the financial impacts of various interventions in different industrial sectors, particularly in Indonesia. In this research, five key types of government interventions are selected amid the pandemic from March 2020 to July 2021, including economic stimulus packages, jobs creation law, Jakarta lockdowns, Ramadan travel restrictions, and free vaccination campaign. Based on an event study methodology, the research reveals that the first economic stimulus package was critical in reviving most sectors following the announcement of the first COVID-19 case in Indonesia. Jakarta lockdowns impacted stock returns negatively in most sectors, but the impacts were relatively insignificant in comparison to other countries in the region. The recurrence of lockdowns in Jakarta had a minor detrimental impact, showing that the market had acclimated to the new normal caused by the COVID-19 pandemic. Additionally, Ramadan travel restrictions caused minor negative impacts on the stock market. Furthermore, the second Ramadan travel restrictions generated a significant reaction from the technology sector. Finally, while free vaccination campaign and job creation law did not significantly boost the stock market, both are believed to result in a positive long-term effect on the country’s economy if appropriately executed. The findings are critical for investors, private companies, and governments to build on recovery action plans for major industrial sectors, allowing the stock market to bounce back quickly and efficiently. As this study limits its analysis to the short-term impact of individual interventions, future studies can examine long-term and combined effects of interventions which could also help policy makers to form effective portfolios of interventions in the event of a pandemic.

**Supplementary Information:**

The online version contains supplementary material available at 10.1007/s43546-022-00312-4.

## Introduction

A contagious disease that started in December 2019, so-called COVID-19, had infected more than 197 million people globally, with 4.2 million deaths as of 31 July 2021 (WHO 2021). The virus attacks the human respiratory system, which leads to a more severe effect on people with underlying medical problems. There have been a few disease outbreaks since the beginning of the twenty-first century when a deadly Severe Acute Respiratory System (SARS) virus tore East Asian countries in 2002 with over 8000 cases. In addition, in early 2009, the United States discovered a new influenza outbreak called Swine Flu (H1N1), which lasted around 19 months and was estimated to cause between 105,000 and 395,000 deaths in over 214 countries. The COVID-19 pandemic is still incomparable to these outbreaks as it utterly changed the way of living, which appeared to cause a domino effect to the world’s economy. The pandemic led to an economic downturn indicated by most countries declaring a recession in the third quarter of 2020. In addition, the stock markets worldwide had significantly lost investor’s confidence due to the uncertainty during the COVID-19 pandemic, which was supported by past experiences (Fan 2003; Bloom et al. 2005). However, although an increase in new cases has shown a negative return in the stock market, each country has faced different impacts from the COVID-19 pandemic. Thus, despite the relatively low mortality rates, this pandemic has created a more significant financial crisis than any other extraordinary event in this century.

**Fig. 1:**
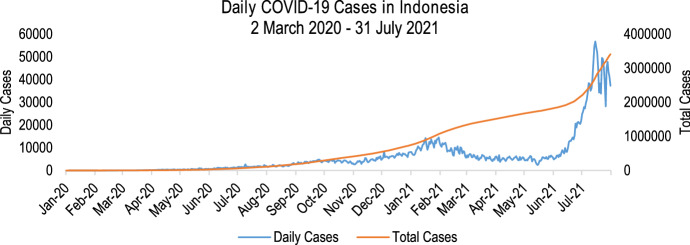
The daily COVID-19 cases in Indonesia from March 2020 to July 2021 (WHO 2021)

This study centres its discussion on the COVID-19 situation in Indonesia, which is selected to reach wider audience from relevant countries in terms of geographic and economic conditions. Geographically, Indonesia is the largest tropical archipelago in the world and it is located in the most populous continent (Cribb and Ford 2009; United Nations 2021). Furthermore, from an economic perspective, Indonesia is also the largest economy in Southeast Asia and the world's seventh largest economy by purchasing power (World Bank 2018). The fourth most populated country declared its first case on 2 March 2020 when the president announced that foreign citizens had transmitted the virus to a local citizen in Jakarta. The COVID-19 cases in Indonesia have proliferated for the past 16 months since March 2020, with 3.4 million cases that caused over 90,000 (WHO 2021). By the end of July 2021, Indonesia has the most active cases in the Southeast Asia region, with over 40,000 daily new cases as shown in Fig. 1.

This study uses the local stock market as the instrument to quantify the impact of the COVID-19 pandemic to the Indonesia’s economy. A stock market is used as it could be utilised as another vital economic indicator beside the country’s GDP. Masoud (2013) supported that the stock market has played a significant role in emerging countries like Indonesia. Hall (2020) also found that the market volatility trend was correlated negatively and significantly with real per capita GDP growth. Therefore, it is believed that any significant impact from government interventions could be detected from the stock market’s short-term movements.

**Fig. 2:**
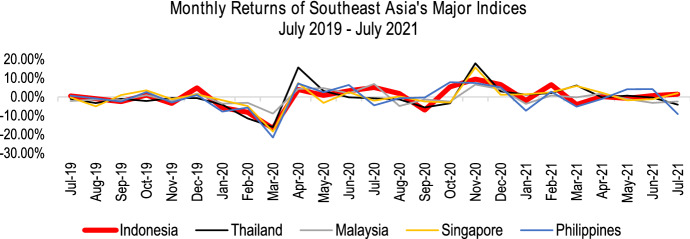
Monthly returns of Southeast Asia’s major indices from July 2019 to July 2021 (Reuters 2021)

Indonesia is selected also due to its relatively stable financial performance over any major indices in the Southeast Asia region as illustrated in Fig. 2. Despite its rapid growth in the number of cases, there is an indication that the government interventions might have played a significant role to recover the stock market’s fall. Although it experienced a significant loss of around 20% in March 2020, the Indonesian Stock Exchange (IDX) has rebounded and performed relatively well at least until July 2021. Hence, the Indonesian Government’s approach during the COVID-19 pandemic could provide an exciting insight into the literature.

The challenging situation has led the Indonesian Government to implement a series of non-pharmaceutical interventions, such as social distancing, travel restriction, and lockdown. In addition, several economic policies, including debt relief, electricity subsidy, tax exemption, and microbusiness support, have also been implemented as it is critical to keep the investors’ positive sentiment, as the country targets an optimistic 5.6–6.2% average GDP growth by 2024 (Reuters 2020). Furthermore, the president had also used political interventions by restructuring his cabinet to strengthen the health and economy ministers to gain more trust from the public.

To understand the effectiveness of the Indonesian government interventions, this study uses an event study methodology to quantify the possibility of short-term indirect impact on each individual sector in the Indonesian Stock Exchange (IDX). Additionally, an event study can analyse numerous similar events to forecast how stock values in a certain sector normally react to a particular event. The methodology has also been applied extensively on both firm specific and industry level in analysing the short-term impact of corporate-related news, financial crisis, marketing strategy, natural disasters and disease outbreaks.

The study contributes to the literature by providing insights into the short-term impact of government interventions during a pandemic. The findings are expected to help investors, regulators, and government understand the short-term impact of government interventions on each industry sector. The insights could also help them create better policies and decisions to avoid a severe economic impact on any future pandemics. The remainder of the study is organised as follows: Sect. 2 reviews the literature related to the research topic, while Sect. 3 discusses the methodology and data collection process for event study analysis. Section 4 presents the results and findings from the analysis. Last, Sect. 5 concludes the study.

## Literature review

### Impact of pandemics on the economy

Many studies have investigated the economic impact of pandemics on regional and worldwide levels. Most of the literature indicated that a pandemic would have a short-term impact on the global economy, with a longer duration for countries identified as the pandemic's epicentres. SARS, the first pandemic of the twenty-first century, began in Hong Kong and infected over 8000 individuals worldwide in 18 months, with a 10% fatality rate. Mackellar (2007) calculated that a relatively small outbreak triggered a 1% drop in China’s GDP and a 0.5% drop in Southeast Asia’s GDP, resulting in a substantial loss of US$ 30 billion. Although Sánchez and Liborio (2012) determined that a relatively insignificant decrease in GDP would not immediately cause a dramatic increase in unemployment rates, they argued that a recession could be incurred with a likelihood of 33%. H1N1 was initially reported in the United States in the spring of 2009, with over 400, 000 confirmed cases by the end of the year (CDC 2019a). The economic impact was difficult to quantify as the economy was still dealing with the effects of the 2008 Global Financial Crisis. However, Barua (2020) suggested that a clear enlightenment might be gained from the tourism business, and they found that Asia’s tourism sector was the quickest to recover from the pandemic by reviving tourist arrivals in the second half of 2009. In addition, Rassy and Smith (2013) analysed the effect on Mexico, which was one of the pandemic’s epicentres, estimated US$ 2.8 billion losses from the tourism industry given the intensity of the H1N1 Kim et al. (2012) estimated that the indirect cost from a pandemic could be 5–10 times more than the direct cost. The 2014 Ebola pandemic was 11 times larger than the previous Ebola outbreaks combined, with over 11,000 deaths by the end of 2016 (CDC 2019b). The outbreak was centred in the West African region, affecting the following three nations in particular: Guinea, Liberia, and Sierra Leone. According to the World Bank report (2016), the three countries lost a total of US$ 2.8 billion. Furthermore, the report also stated that Sierra Leone’s private sector had lost half of its workers, while the unemployment rate in Liberia had climbed by 40%. Interestingly, Ighobor (2014) found that neighbouring countries with no confirmed cases also faced a substantial decline of their GDP by at least 1%, suggesting that tourist and transportation sectors had spillover effects. According to Omoleke et al. (2016), high death rates have resulted in an unprecedented number of persons being pulled from the labour market, resulting in a drastic fall in public consumption.

In comparison to the above-mentioned pandemics, the COVID-19 pandemic has a greater impact. The IMF (International Monetary Fund 2020) reported that the cumulative GDP loss could be around US$ 9 trillion in 2020, with US$ 4 trillion solely contributed from the tourism industry. Additionally, emerging economies were significantly damaged as many declared recessions. Moreover, Jawaid and Garrido (2020) discovered that in the third quarter of 2020, 31 developed economies with a GDP over US$ 200 billion were in a recession. Rose (2021) also predicted that the economic impact of the COVID-19 pandemic would likely to be 30–50 times greater than the 9/11 tragedy.

### Stock market reaction to the COVID-19 pandemic

The adverse reaction in the global stock markets started when WHO declared COVID-19 as a global pandemic, and many governments started to declare its first cases (more details in Appendix A). Most agreed that stock markets underreacted as many showed positive returns within the first few days but suddenly plummeted to their lowest level in the decade. Additionally, firms were showing different reactions to the COVID-19 as SMEs showed significant negative returns while large firms still survived with slight positive returns in the United States (Harjoto Rossi and Paglia 2020). The stock markets’ delayed response might have two possible explanations as follows: different market efficiencies in different countries and the investors’ confidence in the government to deal with the pandemic (Khatatbeh et al. 2020).

**Fig. 3:**
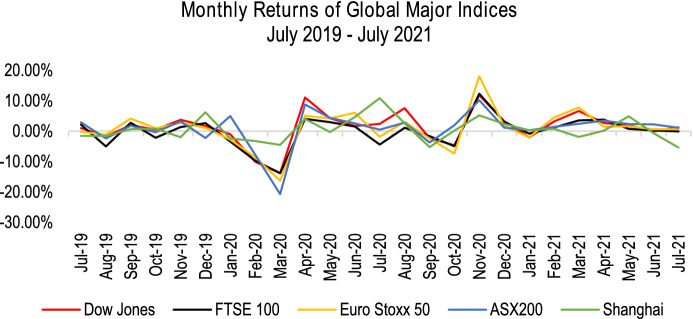
The monthly returns of global indices from July 2019 to July 2021 (Reuters 2021)

The countries with more complex COVID-19 situations had proven to have higher volatility in the stock market. Harjoto et al. (2020a, b) found that emerging markets tend to be more volatile than the developed market with 1% increase in daily cases and deaths caused a 2.37% and 14.94% volatility in the stock market. Fu et al. (2021) examined the global stock markets and concluded that South America was highly exposed to the contagion economic risk, while Asia experienced the most negligible severe risks in the same period. Their study also indicated that the panic caused by the uncertainties led to a severe drop in investor confidence, resulted in a nose-dive response in the global stock market. Gupta et al. (2021) strengthened the claim that the COVID-19 pandemic has negatively impacted all major stock markets in the short term. According to Reuters (2021), the monthly returns for major indices, including Dow Jones, FTSE 100, Euro Stoxx 50, Shanghai and ASX 200 in Fig. 3 showed a relatively stable return before the COVID-19 outbreak but declined sharply from February 2020 to April 2020. Australia’s ASX-200 was one of the most affected indices at the beginning of the pandemic, with a negative 20% return in March 2020. On the other hand, China’s Shanghai index showed a relatively stable return as the country successfully controlled the disease’s transmission rate.

It was discussed that the increase in investor herding behaviour during the COVID-19 epidemic might have contributed to stock market volatility. In general, herding behaviour aggravates fluctuations, resulting in inefficiencies in the stock markets (Blasco et al. 2012). In research of 49 countries around the world, Bouri et al. (2021) discovered a strong connection between herding behaviour and stock market uncertainty, and they discussed that emerging markets exhibit more herding behaviour as a result of more volatility, which is consistent with prior research. Chong et al. (2016) concluded that companies with a high turnover ratio and systematic risk would be swiftly exposed to herding behaviour in their study of China's stock market. In addition, analyst recommendation is one of the factors that played a vital role in causing herding behaviour.

### Government interventions during the COVID-19 pandemic

Disease outbreaks, including the rapid spread of COVID-19, have caused severe human and economic costs to any country globally. Governments were forced to implement strict measures to limit these costs in the short and longer-term. Several studies showed the importance of enforcing the interventions that could change people’s behaviour, which eventually would help reduce the COVID-19 transmission rate, as mentioned in Cowling et al. (2020) study in Hong Kong. Non-pharmaceutical measures, according to many studies, effectively reduced the number of cases reproduced; however, pharmaceutical interventions, such as immunisation, were critical in controlling the COVID-19 pandemic.

We reviewed the impact of the four widely implemented non-pharmaceutical interventions, including contact tracing, social distancing, lockdown, and border restriction in Appendix B. Koh, Naing and Wong (2020) stated that these interventions were implemented by over 142 countries before the 100th COVID-19 cases identified in each country due to their effectiveness in limiting people’s movement and hence reducing the transmission rate of the infectious disease. Governments implemented lockdown as one of the earliest interventions. Lockdown is widely regarded as an effective tool to restrict the transmission rate of infectious diseases, though any lockdown over 120 days is considered ineffective (Koh et al. 2020). Goldstein et al. (2021) suggested that countries should not impose a blind national lockdown which could place the underprivileged people at high risk of unemployment. Instead, a short and strict lockdown would be ideal for emerging countries with a data-driven decision on hospital system situations. When combined with quarantine policies, contact tracking proved to be effective. However, the implementation could be costly for emerging countries, while data protection could be a complicated challenge to overcome by Western countries.

Furthermore, Adekunle et al. (2020) argued that the impact of border restrictions was largely reliant on the underlying situation of the country. Steyn et al. (2021) suggested that Australia and New Zealand had the strongest border restrictions, which were seen to be crucial during the early epidemic, delaying the pandemic by four weeks and giving the government more time to prepare. On the other hand, African countries that enforced border restrictions witnessed an increase in new cases due to a lack of official support to ensure the lives of the people (Emeto et al. 2021). In fact, social distancing was found to be the most effective non-pharmaceutical intervention by significantly reducing the transmission rate by 25% (Li et al. 2020).

### Impact of government interventions on the stock markets

Non-pharmaceutical measures were also applied to reduce COVID-19 transmission. Governments implemented a variety of economic recovery interventions. In doing so, governments previously had enforced direct and indirect stock market interventions. During a crisis, direct interventions had varying results. During the Asian Financial Crisis of 1998, Hong Kong allocated US$15 billion to acquire the Hang Seng Index’s 33 stocks. A positive abnormal return for at least 30 days was found to restore investor confidence successfully, with a short-term spillover impact to other stocks (Su et al. 2001). During the 2008 Financial Crisis, Russia injected the banking system with US$ 150 billion. As a result, the market overreacted on the intervention day, resulting in a significant negative return. Therefore, many governments selected the safer approach with indirect interventions, which had succeeded in many developed countries (Swaine 2008; Murphy 2008). Many studies found that social distancing and lockdown had a short-term negative influence on markets.

We reviewed the impact of three non-pharmaceutical interventions, including lockdown, gathering restrictions, and economic support on the stock markets in Appendix C. Stock markets across the world had plummeted as a result of the lockdown’s implementation. When local governments announced the lockdown, both developed and emerging markets overreacted. Lockdown also had a spillover impact on interconnected countries, as several economies experienced a brief downturn when their neighbours went into lockdown (Eleftheriou and Patsoulis 2020). The cancellation of public events was proven to be the most impactful restriction in causing excessive volatility in the global stock markets (Zaremba et al. 2020). Gathering restrictions were also found to cause high volatility in the global stock markets, with the cancellation of public events believed to be the most impactful restriction (Zaremba et al. 2020). The restriction also significantly reduced the illiquidity situation in America, Europe, and the Middle East emerging economies. However, Asian emerging economies showed no impact on the market’s liquidity (Haroon and Rizvi 2020). Last, economic supports were insignificant in helping the recovery of stock markets. However, several studies concluded that these interventions directly targeted households and not corporations, which generated a relatively small indirect impact. Besides, monetary policies and fiscal policies were impactful in helping stock markets to rebound in all continents. Asian emerging markets were found to have a spillover impact from developed countries’ quantitative easing policies, contributing to an 8% surge on average (Beirne et al. 2021).

## Data and methodology

### Data collection

#### Stock market

The Indonesia Stock Exchange (IDX) was established in 2007 and has shown significant development during the past decade. As of 31 July 2021, the stock market has 746 listed companies, an approximately 50% increase since 2014. However, during the COVID-19 pandemic, the Jakarta Composite Index (JKSE) has reached its 7-year low in March 2020. Nevertheless, JKSE recovered towards the end of 2020 with only a 4.8% loss and outperformed other major indexes in the Southeast Asia region (Maulia 2021).

As a sample for the analysis, we selected the industry sector leaders based on the market capitalisation before the COVID-19 pandemic began. Indonesia, as an emerging market, has a different characteristic from the developed market. As illustrated in Table 1, the financial sector still dominates and has become most of the market in Indonesia, where the technology sector has been on the top list for decades in developed countries like the United States. The stock market data used in this study is considered as a secondary dataset obtained from the Yahoo Finance database from 1 January 2020 to 31 July 2021 to analyse the impact of government interventions selected for this study.

**Table 1:** The Indonesian Stock Exchange (IDX) industry classification (Indonesia Stock Exchange 2021)

Sector name	Sector market capitalisation 2021 (in million USD)	Sector leader	Leader market capitalisation 2020 (in million USD)	Leader market capitalisation 2021 (in million USD)
Financial	178,907	Bank Central Asia	57,965	58,864
Consumer non-cyclical	79,318	Unilever Indonesia	22,230	18,850
Basic material	57,658	Chandra Asri Petrochemical	11,470	12,947
Infrastructure	46,130	Telkom Indonesia	27,592	21,982
Industrial	35,004	Astra International	18,842	17,620
Energy	24,910	Adaro Energy	2872	2739
Consumer cyclical	20,888	Ace Hardware Indonesia	2162	1909
Properties & real estate	19,727	Pollux Properti Indonesia	6418	2344
Healthcare	15,580	Kalbe Farma	4913	4900
Technology	2718	M Cash Integrasi	254	266
Transportation and logistic	1722	Garuda Indonesia	343	536

#### Government interventions

**Fig. 4:**
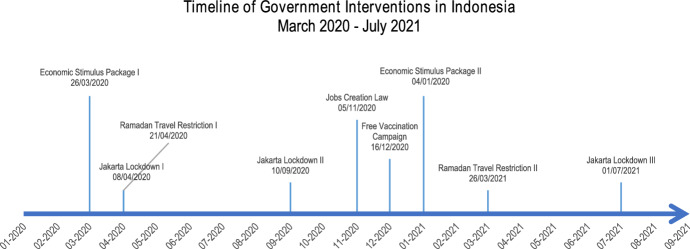
The timeline of five Indonesian government interventions for the analysis

This study selected nine events between March 2020 and July 2021, including two events of economic stimulus packages, one event of jobs creation law, three events of Jakarta lockdowns, two events of Ramadan travel restrictions, and one instance of a free vaccination campaign. The timing for each intervention's announcement is highlighted in Fig. 4. The selection of the nine events is justified in the following sub-sections.

##### Economic stimulus packages

The Indonesian president unveiled the economic stimulus package on 24 March 2020, and it was the first economic policy responded to the COVID-19 pandemic. According to his statement, the economic stimulus package was designed to assist corporate firms in surviving and maintaining people’s purchasing power. The package includes tax breaks for all industries, credit relief for small businesses, and an increase in the amount accessible to Staple Food Card recipients. A total of nine incentives were implemented, resulting in a rise of IDR 405 trillion in the state budget, which is equivalent to US$ 27 billion (Gorbiano and Akhlas, 2020).

This intervention was chosen for various reasons, including the fact that it was the Indonesian government’s first and largest economic intervention during the COVID-19 pandemic. The economic stimulus package also resulted in a substantial increase to the state budget for 2020, which was in place until December 2020. It was believed that the nine incentives provided possibly had an indirect impact on various business sectors, which might be the turning point for stock market downturns. The government had also decided to extend several incentives from the 2020 economic stimulus package into 2021, which was announced by the president on 4 January 2021. The 2021 package focused more on small businesses, impoverished families, and unemployed citizens with a total of IDR 110 trillion allocated, which is reduced than the previous year.

##### Jobs creation law

**Fig. 5:**
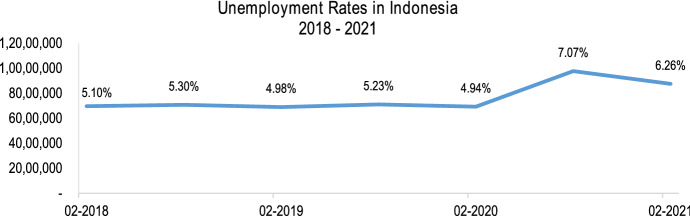
The unemployment rates in Indonesia from February 2018 to February 2021 (BPS Statistics Indonesia 2020)

The global unemployment rates were soaring at the beginning of the COVID-19 pandemic caused by cost-optimisation by most businesses. Indonesia has also experienced a sudden increase within a few months after the pandemic. Figure 5 shows the sharp movement between the first and second quarters of 2020, indicating that at least three million people lost their jobs throughout the period.

During the challenging times, the Indonesian parliament decided to authorise a new law to simplify investment regulations called *Omnibus Law* (Jennings 2020). The complexity of foreign investments in Indonesia has been a long-standing issue, as The World Bank (2020) highlighted the country’s rigid investment regulations as a factor limiting growth. It is also reflected from the 2020 World Bank’s Ease of Business Index that put Indonesia in 73rd place among 190 countries, far behind other Southeast Asia countries. Oxford Business Group (2020) also suggested that the law could provide jobs for six million people who have been left unemployed during the COVID-19 pandemic.

This intervention was selected as one of the most vital and controversial interventions during the COVID-19 pandemic. This is because the international world looked at the law as a brighter future for the Indonesian industries, while the domestic workforce strongly opposed the law due to a weakening in job security. Nevertheless, the *Omnibus Law* was a concrete step for Indonesia’s investments regulations, which could positively impact the country’s economy during the COVID-19 pandemic.

##### Jakarta lockdowns

Jakarta is the Indonesian capital, categorised as a unique province among 33 other provinces in the country. The capital is the most populated city in the Southeast Asia region, with over 10.57 million population at the beginning of 2020 (BPS Statistics Indonesia 2020). In addition, Jakarta is also the headquarters of big companies and where the Indonesia Stock Exchange sits.

**Fig. 6:**
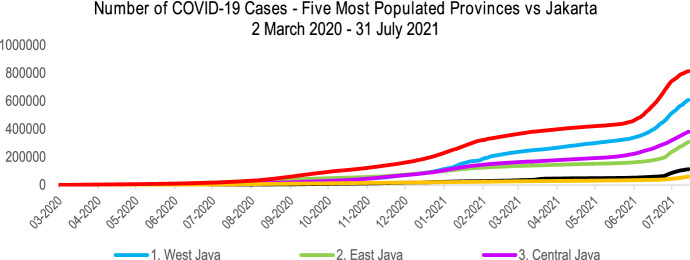
The number of COVID-19 cases in six provinces in Indonesia (Indonesia COVID-19 Response Acceleration Task Force 2021)

Jakarta’s population density is extremely high, over one hundred times the country’s average, with 16,704 people per Km^2^ in 2020 (BPS Statistics Jakarta 2020). Due to the high population density, the COVID-19 cases’ growth in Jakarta was the fastest among other provinces. Figure 6 illustrates the significant difference in the number of COVID-19 cases in Jakarta compared to the five most populated provinces. The six provinces had around 63% of the total cases in Indonesia at the end of 2020, implying that high-population provinces were the pandemic’s epicentres in Indonesia.

This has forced the Indonesian government to impose a regional-level lockdown instead of a national lockdown like other high-population countries like China and Brazil. Jakarta was the first province to impose the lockdown when the governor announced 14 days of large-scale social restrictions on 7 April 2020 when the number of daily new cases surpassed one hundred for two consecutive days (BPS Statistics Jakarta 2020). This was followed by two other lockdowns in September 2020 and July 2021 mainly to avoid public health collapse due to the increase in the new cases.

This intervention was selected for several reasons, considering that Jakarta is the most populated city in Indonesia and headquarter of most businesses in the country. Although Jakarta is the smallest province geographically, it represents the biggest contributor to the country’s GDP with 17.67% in the second quarter of 2020. This indicates that any extreme measurements in Jakarta could impact the country’s economic condition.

##### Ramadan travel restrictions

Indonesia has the largest Muslim population, accounting for approximately 12% of the global Muslim population. The country’s biggest annual event occurs during Ramadan month when tens of millions of people travel to their hometown; a tradition locally called *Mudik,* like Christmas in western countries or the Chinese New Year. This event generally impacts the country’s economy, as people tend to spend more due to the compulsory Ramadan bonus granted to all employees. Moreover, Muslims must pay alms during Ramadan, which creates a massive surge in the money circulation during the season. Hence, the consumer sector typically significantly impacts Ramadan, with an approximately 30% increase in sales (Halimatussadiah 2015).

However, Ramadan travel during the COVID-19 pandemic could result in a massive surge in the new cases as millions of people would travel in any transportation mode. For example, 23 million people travelled domestically during Ramadan in 2019 (Wight 2020). To prevent the disaster, the Indonesian government temporarily banned domestic flights, busses, and ferries for at least 14 days before and after Ramadan Day. These restrictions were imposed in both 2020 and 2021, which have proven to reduce the transmission rate. However, the economic impact was unclear to the country’s economic condition. Hence, this intervention was selected as it would be insightful to explore the economic impact created by the restrictions.

##### Free vaccination campaign

The Indonesian journey with vaccination finally started when the first batch of Sinovac vaccines arrived in Jakarta on 7 December 2020. A week later, President Widodo announced to provide free Covid-19 vaccines, which plays a vital role in other countries to recover their economic condition quickly. For example, the vaccination policy and people’s willingness to get vaccinated gave strong sentiments from the US Stock Market, which is a good indicator of economic healing (US Bank Asset Management Group 2021). In other words, vaccines could help the government to have significant economic growth as people’s movement would be less limited.

Several studies have also found a reasonable hope that vaccines should help the world stop the pandemic. For example, Powell (2021) concluded that vaccines are created to establish herd immunity, which can be achieved when 50–60% population is vaccinated regardless of the virus mutations, as it would still have the same structure. Therefore, this intervention was selected as it might be the booster for the stock market to gain more trust from the investors and started to grow strongly towards 2021.

### Methodology

The study used the event study methodology, which has been widely utilised to assess the valuation impacts of extraordinary corporate actions (more details in Appendix D). In addition, event study has revealed vital information about how an industrial sector is likely to react in a short-term to a given extraordinary event, such as natural disaster, disease outbreaks, and geopolitical issues. A short-term analysis is critical for the stock market since an extraordinary event might alter investor behaviour and the entire market environment.

**Fig. 7:**
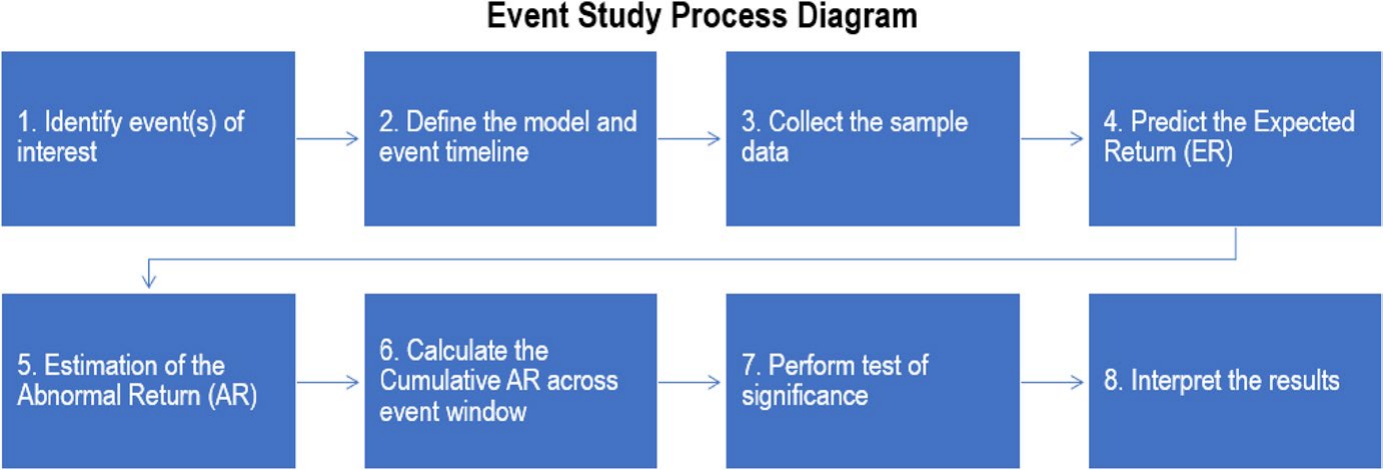
The process diagram for event study analysis

The approach examines the stock price’s response around the announcement by looking at the stock returns first introduced by Dolley (1933). Moreover, event study with known event dates has a relatively statistical solid power to support the result, which is required to understand the short-term impact during unprecedented events like the COVID-19 pandemic. Although Dyckman et al. (1984) pointed out several problems of daily return analysis, such as nonsynchronous trading and biased estimation, Brown and Warner (1985) concluded that the potential problems with daily returns are unimportant easily corrected in the standard event study. Figure 7 illustrates the process taken by this study to perform the event study analysis, and the key steps are described in the following subsections.

#### Model

Three event study models were commonly used in previous studies, namely constant mean return, market model, and capital asset pricing model (CAPM). The main difference among these models lies in the way of calculating the expected returns and abnormal returns. The constant mean return model has the simplest from by subtracting the stock return with the simple mean return, but Brown and Warner (1985) criticised that the method does not consider the abnormal return in reflection to the stock market condition. The market model and CAPM are the most popular yet similar methods in practice. The difference is that the CAPM imposes an additional restriction (e.g., intercept equals the risk-free rate). Due to the added restriction, the variance of error terms in CAPM is generally more significant than the market model (MacKinlay 1997). Consequently, a significant variance of error leads to a less powerful test for the result than the market model. This study uses the seminal market model introduced by Scholes and Williams (1977).

#### Timeline

**Fig. 8:**
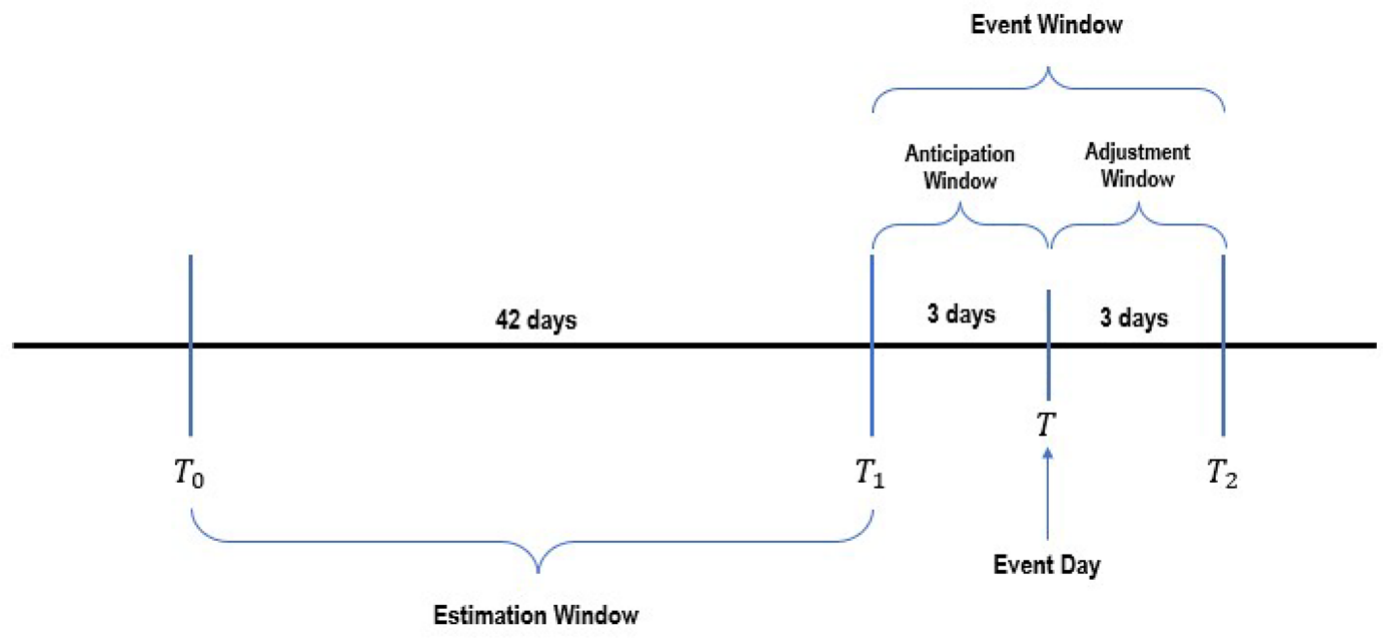
Illustration of the event window timeline used in the paper

There are two crucial parameters that determine the analysis’s outcome in the event study, namely the estimation window and the event window. This study used 42 days of the estimation window, equivalent to 2 months of trading days, as the events analysed happen within a short period of 16 months. Furthermore, we used an event window of 7 days, which centres symmetrically around the event day, as shown in Fig. 8, and a similar approach has been taken by previous literature (Bash and Alsaifi 2019; Buigut and Kapar 2020). A short event window is also applied to prevent overlapping event window periods, as some events in the analysis are only a few weeks away. Further, the small period within the event window, the anticipation window and the adjustment window are used to capture the short-term abnormal returns before and after the event day. In contrast, a long event window could reduce the statistical power of analysis but suffer from essential limitations (Brown and Warner 1985).

#### Estimation of expected return

The market model assumes that the asset returns are given by the following:

1 $${R}_{i,t }=E\left[{R}_{i,t }| {X}_{t}\right]+{\varepsilon }_{i,t},$$

where ${R}_{i,t}$ represents the return for each company *i* on day *t,* which belongs to the estimation window, while the expected return is established as follows:

2 $$E\left[{R}_{i,t }\right| {X}_{t}]= {\alpha }_{i}+{\beta }_{i} {R}_{m,t}.$$

The ${R}_{m,t}$ represents the market portfolio’s return, and the linear specification of the model arises from the assumed joint normality of returns. The market portfolio used is the Jakarta Composite Index (JKSE), the composite index for IDX. The market model also assumes that ${\varepsilon }_{i,t}$ changes related to the return on the market portfolio iare removed as follows:

3 $$E{[\varepsilon }_{i,t}]= 0.$$

#### Estimation of abnormal return

This study used Buy and Hold Abnormal Returns (BHAR), which employs a geometric method to calculate abnormal returns. The BHAR approach was used as several economists such as Ritter (1991) and Lyons (1999) have argued that CAR is not appealing from the economic perspective. The CAR approach could lead to biases due to the continuous compound rate of appreciation. The calculation of BHAR is established as follows:

4 $${\mathrm{BHAR}}_{t;t+k}^{i}={\Pi }_{k} \left(1+{\mathrm{AR}}_{i,t+k}\right),$$

where *i* represents each company in the analysis, *t* represents the event window start date, and *k* represents the duration of the event window. Furthermore, the calculation of Abnormal Return (AR) is as follows:

5 $${AR}_{i,t}= {R}_{i,t} - E\left[{R}_{i,t }\right| {X}_{t}].$$

#### Test procedure

The parametric test is the only approach to test the null hypothesis for the event study that analyses multiple individual events’ impact (Boehmer 1991). We used a statistical test to determine whether enough evidence exists to reject a hypothesis about the process. The following hypothesis testing is adapted to the parametric test:

$${H}_{0}:\mathrm{Event has no impact to the stock market return}$$

$${H}_{1}:\mathrm{Event has impact to the stock market return}$$

The statistical test is formulated as follows:

6 $${t}_{\mathrm{BHAR} }= \frac{{\mathrm{BHAR}}_{i,t}}{\frac{\sigma \left({\mathrm{BHAR}}_{i ,t}\right)}{\sqrt{n}} }.$$

The ${t}_{\mathrm{BHAR}}$ represents the *t*-score of the BHAR for each company at different event windows and ${\sigma }_{BHAR}$ is the standard deviation of the BHAR for the estimation window.

#### Implementation

This paper used an analytical approach to answer the research questions, mainly for the event study analysis. Open-source packages in Python are used to automate the data extraction process, calculate the daily returns, estimate the abnormal returns, and validate the results.

## Results and discussion

### Preliminary analysis

#### Stock data

To demonstrate the unique circumstances surrounding the COVID-19 pandemic, descriptive statistics for the years 2019–2021 are presented for comparison. A majority of industries experienced a fall in the average stock price, as demonstrated by the Jakarta Composite Index (JKSE), which experienced a roughly 16% decline in the average index price in 2020 (Tables 2 and 3). However, two of the eleven sectors, basic material and property, experienced a significant increase in the average stock price, nearly doubling the previous year.

**Table 2:** The summary statistics of the Indonesian Stock Exchange in 2019

Sector name	Sector leader	Mean	Standard deviation	Minimum	Maximum	Range
Financials	BBCA	28,247.735	1844.932	24,727.080	32,643.193	7916.113
Consumer non-cyclical	UNVR	8624.220	436.600	7754.254	9324.677	1570.422
Basic materials	TPIA	6834.609	1852.691	4658.167	10,437.050	5778.882
Infrastructures	TLKM	3628.245	206.348	3136.425	4049.256	912.831
Industrials	ASII	6684.470	462.969	5941.070	7795.317	1854.247
Energy	ADRO	1131.249	95.345	888.623	1441.834	553.211
Consumer cyclical	SCMA	1472.038	235.150	1058.400	1930.277	871.877
Properties and real estate	POLL	3761.837	3169.101	1100.000	11,150.000	10,050.000
Healthcare	KLBF	1473.208	89.058	1191.469	1628.212	436.743
Technology	MCAS	3398.898	264.622	2680.000	3930.000	1250.000
Transportation and logistic	GIAA	473.988	76.073	290.000	630.000	340.000
**Composite Index**	**JKSE**	**6296.088**	**149.052**	**5826.868**	**6547.877**	**721.009**

The bold values are for Jakarta Composite Index, and the non-bold values are eleven individual industrial sectors

**Table 3:** The summary statistics of the Indonesian Stock Exchange in 2020

Sector name	Sector leader	Mean	Standard deviation	Minimum	Maximum	Range
Financials	BBCA	29,442.582	2836.985	21,407.750	34,269.617	12,861.867
Consumer non-cyclical	UNVR	7539.756	495.132	5415.674	8219.363	2803.689
Basic materials	TPIA	7770.834	1095.232	5206.129	10,288.304	5082.174
Infrastructures	TLKM	2968.487	300.801	2373.389	3650.671	1277.282
Industrials	ASII	5007.882	886.454	3108.480	6823.493	3715.012
Energy	ADRO	1085.334	171.415	587.663	1486.752	899.090
Consumer cyclical	SCMA	1231.070	328.453	635.000	2320.000	1685.000
Properties and real estate	POLL	7297.058	2694.155	3600.000	11,725.000	8125.000
Healthcare	KLBF	1404.717	160.320	833.375	1604.126	770.751
Technology	MCAS	1862.654	647.980	645.000	3990.000	3345.000
Transportation and logistic	GIAA	287.798	88.320	150.000	498.000	348.000
**Composite Index**	**JKSE**	**5253.297**	**553.609**	**3937.632**	**6325.406**	**2387.774**

The bold values are for Jakarta Composite Index, and the non-bold values are eleven individual industrial sectors

Table 4 summarises the statistics data for 2021, which is considerably different from the previous year. The market has generally recovered, with an average index price increase of 16% until July 2021. Ten of the eleven industries also had strong growth, except for the property sector, which experienced a substantial decline. Although there are still a few months remaining before the end of 2021, these figures indicate that the market has begun to recover, despite Indonesia continuing to have the most cases in Southeast Asia as of 31 July 2021.

**Table 4:** The summary statistics of the Indonesian Stock Exchange in 2021 (until 30 July 2021)

Sector name	Sector leader	Mean	Standard deviation	Minimum	Maximum	Range
Financials	BBCA	28,247.735	1844.932	24,727.080	32,643.193	7916.113
Consumer non-cyclical	UNVR	8624.220	436.600	7754.254	9324.677	1570.422
Basic materials	TPIA	6834.609	1852.691	4658.167	10,437.050	5778.882
Infrastructures	TLKM	3628.245	206.348	3136.425	4049.256	912.831
Industrials	ASII	6684.470	462.969	5941.070	7795.317	1854.247
Energy	ADRO	1131.249	95.345	888.623	1441.834	553.211
Consumer cyclical	SCMA	1472.038	235.150	1058.400	1930.277	871.877
Properties and real estate	POLL	3761.837	3169.101	1100.000	11,150.000	10,050.000
Healthcare	KLBF	1473.208	89.058	1191.469	1628.212	436.743
Technology	MCAS	3398.898	264.622	2680.000	3930.000	1250.000
Transportation and logistic	GIAA	473.988	76.073	290.000	630.000	340.000
**Composite Index**	**JKSE**	**6296.088**	**149.052**	**5826.868**	**6547.877**	**721.009**

The bold values are for Jakarta Composite Index, and the non-bold values are eleven individual industrial sectors

The range is a crude measure of the spread in stock prices, and it indicates that the range for the majority of sector leaders increased significantly in 2020 compared to 2019. The range, however, is subject to outliers that occur under an extreme COVID-19 situation. In comparison, the standard deviation is a more accurate tool for detecting outliers in a normal distribution. This method showed a substantial increase from 2019, indicating that the stock market saw much higher volatility returns as the dispersion of company prices compared to their average increased dramatically. Additionally, it implies that the stock market became a riskier investment during the COVID-19 pandemic in 2020.

On the other hand, both measures have been significantly reduced for the first half of 2021. The composite index’s range and standard deviation have decreased by 72% compared to the previous year. Additionally, nine of eleven industry sectors followed the movement of the composite index. This could indicate that the market has grown less risky and has rebounded from its early 2020 collapse. Furthermore, low volatility attracts additional investors, signalling the prospect of significant growth during the pandemic.

#### Government interventions

**Fig. 9:**
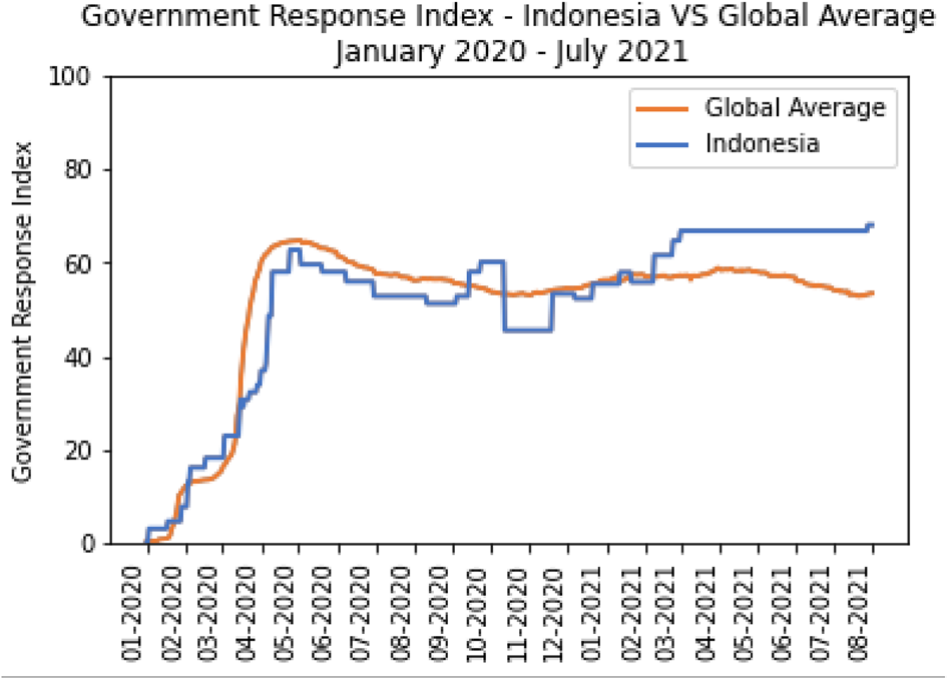
The comparison of Government Overall Index between Indonesia and the Global average

Hale et al. (2021) developed a government intervention tracer that covers 23 different types of government interventions, including containment and closure policies, economic policies, health system policies, and vaccination policies. In this research, we focussed primarily on the following two indices: overall government response and stringency. We compared and analysed the daily global average government response index to the Indonesian daily index in Fig. 9. At the start of the pandemic, the Indonesian government responded more adequately than the global average, implementing at least three critical interventions. However, this condition did not last long, as it continued below the global average for the remainder of 2020. In contrast, the Indonesian government has responded more positively from the beginning of 2021, whereas the global average has declined since May 2021.

**Fig. 10:**
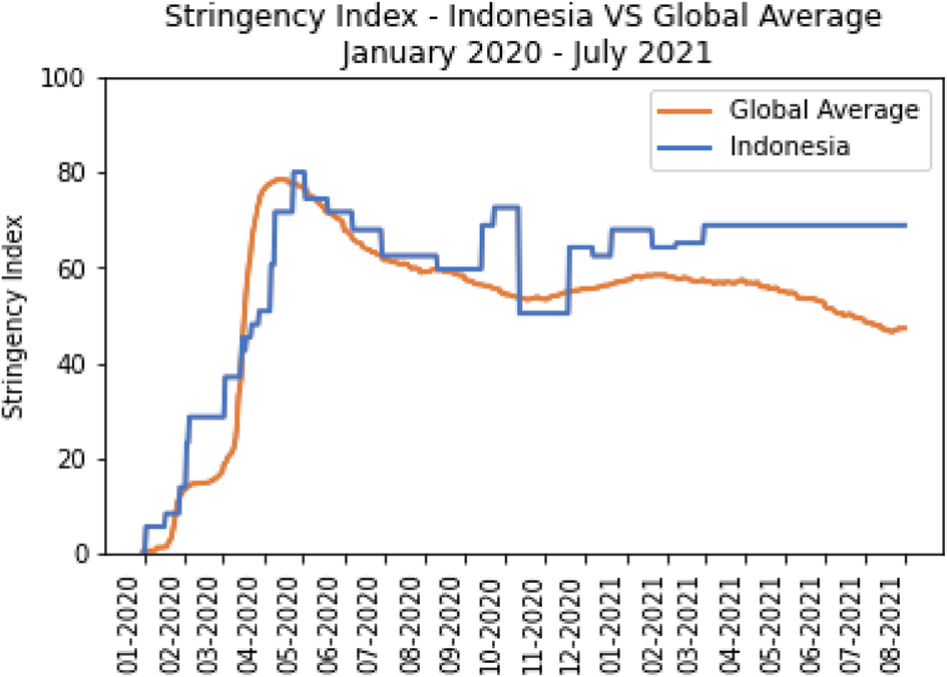
The comparison of Stringency Index between Indonesia and the Global average

The stringency index provided a similar trend to the government response index in Fig. 10. Global average stringency increased rapidly from March to May 2020 but then stabilised until early 2021. Henceforth, the worldwide average has been close to or below the index level of 60. In comparison, Indonesia showed its most stringent condition in May 2020, when the government planned to suspend all modes of public transportation for more than 14 days, with an index was above 80. Although the trend had been downward for several months, it started to rise again in September due to Jakarta's second lockdown. Thus, throughout the COVID-19 pandemic, it is safe to say that Indonesia had implemented tighter restrictions than the global average.

### Event study analysis

The results for each intervention type are presented and discussed in the following sub-sections, with the individual parametric test results presented in Appendix E.

#### Economic stimulus packages

The impact made by the first economic stimulus package was believed to be highly significant for several sectors (Table 5). It is evident that, on the announcement day, the stock prices for financial, consumer non-cyclical, and consumer cyclical sectors were significantly improved. At the same time, infrastructure and healthcare were also increased to a certain extent. In contrast, significant adverse reactions were shown by the stock prices of basic material and property sectors.

**Table 5:** Results of the impact of economic stimulus package on 26 March 2020

Sector name	Sector leader	Abnormal returns			
		Anticipation window (− 3, 0), (%)	Event day (0, 0), (%)	Adjustment window (0, 3), (%)	Event window (− 3, 3), (%)
Financial	BBCA	0.73	6.44**	− 0.82	6.33
Consumer non-cyclical	UNVR	4.67	9.56**	2.46	17.5**
Basic material	TPIA	7.02	− 8.17**	− 15.92**	− 17.37*
Infrastructure	TLKM	4.49	2.37	0.14	7.11
Industrial	ASII	− 9.91**	− 3.55	1.92	− 11.44*
Energy	ADRO	41.05**	− 4.64	− 5.34	27.32**
Consumer cyclical	ACES	− 0.17	8.07**	− 1.48	6.29
Properties and real estate	POLL	− 0.6	− 4.99**	16.50**	10.03
Healthcare	KLBF	− 6.96	0.21	21.87**	13.64*
Technology	MCAS	− 13.12	− 1.52	− 10.73*	− 23.62**
Transportation and logistic	GIAA	− 3.58	− 0.64	2.52	− 1.79

Note: ‘**’ means significance at the 5% level and ‘*’ means significance at the 10% level

It is believed that the financial sector’s reaction was due to the inclusion of financial system stability policies in the package. The policies grant authority to five vital government bodies to establish steps on handling financial stability matters by formulating government support such as short-term liquidity loans and financing on the sharia principle to all financial institutions (Molina and Ramadhan 2020). The stability policies were implemented to avoid a serious banking crisis in 1998 when half of the private banks collapsed due to the government’s unpreparedness in handling a crisis (Fane and McLeod 2002).

Moreover, additional funds for staple food card beneficiaries and pre-employment card holders significantly impacted both consumer sectors’ stock prices. According to Fang (2021), additional funds could stimulate the subsidy by the government to generate more consumption. Liu et al. (2020) strengthened the theory by finding that a consumption coupon of RMB 1 can drive excess spending of RMB 3.4 to RMB 5.8 at the beginning of the pandemic in China. Thus, these findings could be an essential variable that increased the market’s confidence in both consumers sectors on the announcement day.

Additionally, the property sector signalled an under-reaction but rebounded with a positive 16.50% abnormal return during the adjustment window. The under-reaction to stock-related news could be due to an anchoring bias or slow information diffusion (Lansdorp and Jellema 2013). On the other hanfd, basic materials, industrials, and technology significantly suffered. We believed that the corporate tax reduction for 2020 and 2021 is the only policy that directly impacts these sectors.

The impact made by the second economic stimulus package on stock markets was not significant compared to the first one (Table 6). The announcement day has resulted in diverse reactions, where six sectors reacted negatively and five sectors reacted positively. The second economy package was different as it was more focused on income support and debt relief. Ashraf (2020) found that the insignificant impact caused by the package was only directed to households and did not directly impact corporations. Additionally, the results showed that consumer non-cyclical overreacted on the announcement day, followed by a significant negative abnormal return of 3.10% in the subsequent days, while basic material showed an under-reaction with a significant increase of 4.95% to the stock prices in the same period. Besides, basic material showed an underreaction with a significant increase of 4.95% to the stock prices in the same period. Although the impact was not significant, we believed that the announcement was still made at the right time. The market could lead to high volatility if the government did not announce the continuation of several economic stimulus at the beginning of 2021.

**Table 6:** Results of the impact of the second economic stimulus package on 4 January 2021

Sector name	Sector leader	Abnormal returns			
		Anticipation window (− 3, 0), (%)	Event day (0, 0), (%)	Adjustment window (0, 3), (%)	Event window (− 3, 3), (%)
Financial	BBCA	1.52	− 1.43	1.25	1.33
Consumer non-cyclical	UNVR	0.09	1.16	− 3.10*	− 1.89
Basic material	TPIA	− 1.78	2.08	4.95**	5.23
Infrastructure	TLKM	1.16	1.75	− 3.81	− 0.99
Industrial	ASII	1.32	0.85	− 3.12	− 1.01
Energy	ADRO	− 3.45	− 0.98	− 4.46	− 8.66
Consumer cyclical	ACES	− 2.68	− 3.04	4.30	− 1.58
Properties and real estate	POLL	− 3.44	− 2.85	− 6.01	− 11.83
Healthcare	KLBF	2.22	− 0.97	1.24	2.48
Technology	MCAS	2.79	3.79	4.74	11.74
Transportation and logistic	GIAA	− 9.61	− 3.23	− 5.45	− 17.30

Note: ‘**’ means significance at the 5% level and ‘*’ means significance at the 10% level

In addition, we also believed that the Indonesian government had also successfully maximised its capabilities. Table 7 shows the percentage of total COVID-19 economic support to the respective country’s GDP in 2020. Indonesia only spent 2.6% of its GDP to support the economy during the COVID-19 pandemic, which was only higher than China and Taiwan. From the relatively low budget, the Indonesian government successfully targeted the support’s recipients, which reflected by the stock market reaction. Moreover, a low cost of living could also contribute heavily to the economic stimulus package. As an illustration, the average meal price in Jakarta is US$2.51 compared to Singapore and Hong Kong, with US$9.84 and US$7.70, respectively (Numbeo 2021). Therefore, Singapore and Hong Kong must have provided direct household support over US$1000 a month for vulnerable groups. In comparison, the Indonesian government only provided additional support of less than US$100 a month.

**Table 7:** Total economic support measures across Asia Pacific in 2020 (Oxford Economics, 2020)

Country	Economic support as % of GDP in 2020
China	1.30
Taiwan	1.90
Indonesia	2.60
South Korea	7.40
Hong Kong	10.00
Thailand	11.40
Singapore	12.00
Malaysia	17.20

#### Jobs creation law

The Authorisation of the *Omnibus Law* elicited negative sentiments from most Indonesia’s sectors, with eight out of eleven sectors reacting negatively on the day of the announcement (Table 8). By comparison, the infrastructure and financial sectors generated strong positive abnormal returns of 4.17% and 1.92%, respectively. Moreover, under-reaction was detected as the stock price for the transportation sector rose significantly by 19.31% in the subsequent days after the announcement. Overall, most sectors suffered a short-term negative impact during the event window, where the healthcare sector plunged significantly by 9.07% and transportation rose strongly by 17.15%.

**Table 8:** Results of the impact of Jobs creation law on 5 November 2020

Sector name	Sector leader	Abnormal returns			
		Anticipation window (− 3,0), (%)	Event day (0,0), (%)	Adjustment window (0,3), (%)	Event window (− 3,3), (%)
Financial	BBCA	1.51	1.92*	0.84	4.33
Consumer non-cyclical	UNVR	0.11	0.2	− 2.46	− 2.15
Basic material	TPIA	− 1.42	− 0.61	0.44	− 1.59
Infrastructure	TLKM	− 0.54	4.17**	− 0.52	3.07
Industrial	ASII	− 0.46	− 2.64	− 1.45	− 4.49
Energy	ADRO	− 1.06	− 1.59	− 5.48	− 7.98
Consumer cyclical	ACES	0.98	− 2.83	− 1.04	− 2.9
Properties and real estate	POLL	− 21.14	− 0.87	− 20.16	− 37.58
Healthcare	KLBF	− 4.11	− 0.49	− 4.72	− 9.07**
Technology	MCAS	− 2.94	− 4.97	− 2.37	− 9.95
Transportation and logistic	GIAA	− 0.12	− 1.7	19.31**	17.15**

Note: ‘**’ means significance at the 5% level and ‘*’ means significance at the 10% level

The early reaction of the financial and infrastructure sectors was expected, given our belief that the Omnibus Law would directly influence those sectors. For instance, the financial sector might save high operational costs because of worker protections. On the other hand, enterprises involved in telecommunications infrastructure may benefit from the law as it enables them to share their infrastructure, generating enormous synergy and accelerating the sector’s growth.

Despite successfully lowering unemployment rates in the hope of accelerating economic growth during COVID-19, the stock market reacted unexpectedly. Before the event, various Non-Governmental Organisations (NGOs) publicly criticised the law on social media platforms and on national televisions. Valle-Cruz et al. (2022) proved that social media transmission via Twitter directly affected the indices' behaviour, particularly in Indonesia, which has the fourth-largest Twitter user base in July 2021 with 15.7 million active users. Additionally, they determined that the drop in market values during the COVID-19 pandemic was more severe than during the H1N1 pandemic, owing to the abundance of speculation and rumours about the virus. Furthermore, thousands of people protested in October 2020 to express their dissatisfaction with the new law, which could temper market enthusiasm, as happened in the United States when the government’s proposal was encountered with widespread scepticism on its implementation (Randall 2021).

While the Omnibus Law was insignificant in the short term, we believed that the new laws would positively affect several sectors over time. The property sector should be a clear winner, as the Omnibus Law simplified the land permission process, which had been a major obstacle for decades. The simplified foreign property ownership restrictions would also entice many international investors to invest in the Indonesian market. Additionally, the energy and basic material sectors would greatly benefit from a 0% royalty on value-added to raw materials. The industrial sector may also benefit from simplified foreign direct investment (FDI), which could boost the sector’s long-term growth. To summarise, the *Omnibus Law* represents a promising start for Indonesia’s future investment, while implementation would be critical.

#### Jakarta lockdowns

Indonesia was the last major country in Southeast Asia to impose a lockdown. The announcement of Jakarta’s first lockdown appeared to cause adverse reactions from most sectors in Indonesia (Table 9). The stock market showed relatively normal returns on the announcement day of the first lockdown, with only the financial sector, reacting significantly positive. Financials and consumer non-cyclical sectors were underreacted on the announcement day as both plummeted significantly during the adjustment window. At the same time, properties continued to plunge significantly with a negative 14.45% of abnormal return. Overall, eight sectors suffered negative abnormal returns during the event window, with properties significantly suffered while basic materials and transportations were positively affected.

**Table 9:** Results of the impact of the first lockdown in Jakarta on 8 April 2020

Sector name	Sector leader	Abnormal returns			
		Anticipation window (− 3, 0), (%)	Event day (0, 0), (%)	Adjustment window (0, 3), (%)	Event window (− 3, 3), (%)
Financial	BBCA	− 2.87	3.07*	− 5.12	− 5.01
Consumer non-cyclical	UNVR	− 8.75*	1.64	− 4.15	− 11.11
Basic material	TPIA	25.02**	3.01	4.68	34.8**
Infrastructure	TLKM	− 4.22	− 0.04	1.13	− 3.18
Industrial	ASII	− 3.28	− 0.68	1.22	− 2.77
Energy	ADRO	− 0.43	− 2.55	− 2.96	− 5.84
Consumer cyclical	ACES	2.18	− 3.69	− 2.91	− 4.45
Properties and real estate	POLL	− 9.31	− 4.83	− 14.45**	− 26.17**
Healthcare	KLBF	− 11.37*	− 3.72	0.25	− 14.46
Technology	MCAS	0.60	0.28	7.94	8.89
Transportation and logistic	GIAA	13.99**	− 1.22	3.24	16.24**

Note: ‘**’ means significance at the 5% level and ‘*’ means significance at the 10% level

The first lockdown had such a minor detrimental impact that could be due to the Indonesian government’s delay in enforcing one. According to Ozili and Arun (2020), the first regional restriction had a more significant impact than the first national restriction. In other words, when the Philippines announced its first lockdown, the stock market in Indonesia may have already experienced a spillover effect. Furthermore, an introduction of lockdown measures has also been linked to undesirable reactions known as overreaction. Within a few days after the release, most industries showed overreaction, suggesting a delay in absorbing unusual news. Liew and Puah (2020) also verified that various industries experienced varied levels of lockdown based on their market conditions and business nature.

The property sector was also predicted to suffer substantial losses during the initial lockdown. This suggests that the debt-relief scheme included in the economic stimulus package was insufficient to convince the market. Furthermore, transportation sectors reacted negatively to the statement, which was expected given that logistic companies were permitted to operate regularly during the lockdown. Instead, the sector should have received more demands due to the drastic increase in transactions from online marketplaces, as offline stores were forced to halt its operations during lockdown.

The second lockdown in Jakarta elicited a range of responses from most sectors, with less severe adverse effects (Table 10). Though six sectors saw abnormal returns on the announcement, the impact was substantially negative for consumer non-cyclical and basic materials. Additionally, the stock market experienced under-reaction, as basic materials and technology stocks rose 5.75% and 21.89%, respectively, in the days following the announcement. The technology sector’s response was expected, as most enterprises, organisations, and schools must rely extensively on technology companies because of the lockdown restrictions. Additionally, the basic materials sector has recovered globally and was expected to be immune to COVID-19 by the second half of 2020 due to a resurgence in demand from China as the largest global importer (Barman 2020). Overall, the announcement resulted in five of eleven sectors suffering negative abnormal returns, while the remaining nine experienced positive abnormal returns.

**Table 10:** Results of the impact of the second lockdown in Jakarta on 10 September 2020

Sector name	Sector leader	Abnormal returns			
		Anticipation window (− 3, 0), (%)	Event day (0, 0), (%)	Adjustment window (0, 3), (%)	Event window (− 3, 3), (%)
Financial	BBCA	− 0.41	− 1.48	− 3.93*	− 5.73*
Consumer non-cyclical	UNVR	− 1.57	− 1.95*	0.01	− 3.48
Basic material	TPIA	− 0.30	− 4.29**	5.75*	0.9
Infrastructure	TLKM	0.70	1.89	1.51	4.16
Industrial	ASII	0.38	1.74	− 1.42	0.68
Energy	ADRO	1.98	1.29	− 1.21	2.05
Consumer cyclical	ACES	0.49	− 1.50	0.88	− 0.15
Properties and real estate	POLL	53.33**	2.96	68.64**	166.24**
Healthcare	KLBF	− 1.19	− 1.34	1.19	− 1.36
Technology	MCAS	− 10.38	− 1.88	21.89**	7.18
Transportation and logistic	GIAA	1.71	0.00	− 2.55	− 0.88

Note: ‘**’ means significance at the 5% level and ‘*’ means significance at the 10% level

The repetition of the same interventions appeared to have had a stabilising effect on the stock market, particularly proven by the third lockdown in Jakarta (Table 11). Our results aligned with Scherf et al. (2022), who concluded that multiple lockdown restrictions generally caused smaller negative returns than the first one. On the announcement day, consumer non-cyclical and property substantially impacted stock prices, gaining 5.78% and 23.79%, respectively. Additionally, a substantial under-reaction was detected in the consumer cyclical sector, which increased by 18.85% during the adjustment window. Six sectors, in aggregate, responded negatively to the announcement, with the infrastructure sector suffering the most, with an abnormally negative return of 8.43%. In comparison, five sectors experienced positive reactions, with consumer cyclical and property experienced considerable increases in stock prices of 12.42% and 35.87%, respectively.

**Table 11:** Results of the impact of the third lockdown in Jakarta on 1 July 2021

Sector name	Sector leader	Abnormal returns			
		Anticipation window (− 3, 0), (%)	Event day (0, 0), (%)	Adjustment window (0, 3), (%)	Event window (− 3, 3), (%)
Financial	BBCA	− 2.19	− 0.28	− 0.07	− 2.54
Consumer non-cyclical	UNVR	0.6	5.78**	− 3.45	2.75
Basic material	TPIA	− 1.28	− 0.27	− 4.06	− 5.55
Infrastructure	TLKM	− 2.71	− 1.73	− 4.22	− 8.43**
Industrial	ASII	1.4	1.48	− 2.85	− 0.03
Energy	ADRO	− 6.61	− 0.69	5.29	− 2.35
Consumer cyclical	ACES	− 5.35	− 0.07	18.85**	12.42**
Properties & real estate	POLL	2.96	23.79**	6.6	35.87*
Healthcare	KLBF	4.05	0.06	− 1.86	2.18
Technology	MCAS	3.54	− 3.99	− 4.6	− 5.17
Transportation and logistic	GIAA	3.17	0.65	2.21	6.13r

Note: ‘**’ means significance at the 5% level and ‘*’ means significance at the 10% level

#### Ramadan travel restrictions

China enforced its first nationwide travel restriction during the 2020 Lunar New Year. Huo and Qiu (2020) discovered a significant negative impact on the Chinese stock market during the period, with 22 out of 28 sectors experiencing negative abnormal results. Similarly, Indonesia enforced four-week nationwide travel restrictions during Ramadan in 2020 and 2021, but it was found to have a minor impact than China.

On the announcement day, only four sectors suffered a negative abnormal return, while the remainder experienced a positive abnormal return (Table 12). Additionally, throughout the anticipation window, a similar trend was seen with no substantial abnormal returns. However, the consumer cyclical sector underreacted on the announcement day, as indicated by a significant increase of 8.64% within the adjustment window, while properties underreacted by 11.43%. In general, the announcement had no discernible effect on any sector in Indonesia. Most sectors demonstrated insignificantly positive reaction, apart from the financial, energy, property, and transportation sectors, which suffer severe abnormal returns throughout the event window. Additionally, the overall positive returns demonstrated by both consumer sectors indicated that Ramadan spending was resilient to the COVID-19 pandemic.

**Table 12:** Results of the impact of travel restrictions during Ramadan on 21 April 2020

Sector name	Sector leader	Abnormal returns			
		Anticipation window (− 3, 0), (%)	Event day (0, 0), (%)	Adjustment window (0, 3), (%)	Event window (− 3, 3), (%)
Financial	BBCA	− 1.58	− 0.31	− 4.95	− 6.74
Consumer non-cyclical	UNVR	− 0.21	0.38	6.42	6.59
Basic material	TPIA	3.35	1.67	9.67	15.24
Infrastructure	TLKM	1.01	0.02	− 0.03	1.00
Industrial	ASII	− 0.34	0.22	0.68	0.56
Energy	ADRO	− 4.93	− 3.56	− 6.81	− 14.55
Consumer cyclical	ACES	3.89	− 0.76	8.64*	12.01
Properties & real estate	POLL	3.51	1.54	− 11.43	− 6.90
Healthcare	KLBF	− 0.77	2.23	2.61	4.10
Technology	MCAS	− 0.16	0.71	4.64	5.22
Transportation and logistic	GIAA	− 0.11	− 2.34	− 7.14	− 9.41

Note: ‘**’ means significance at the 5% level and ‘*’ means significance at the 10% level

We believed that information leakage was critical in preventing a short-term stock market collapse. For instance, a few days before the announcement, government officials talked about the possibility of travel restrictions in an open public forum. Journalists spread the rumours over multiple media resulting in a higher anticipated reaction from the stock market. Brunnermeier (2005) supported the notion that information leakage makes price processes more informative in the short run, indicating that information leakage frequently helped investors in managing expectations, hence stabilising the stock price in the short term. However, the study also discovered that information leakage might eventually diminish information efficiency. Additionally, the relatively positive responses from most sectors may reflect the market's confidence in the government's commitment to reduce the COVID-19 transmission rate.

A less severe impact because of subsequent measures is also demonstrated by the second nationwide travel ban in 2021 (Table 13). Only the technology sector reacted positively on the announcement, with an 11.64% gain in its stock price. Additionally, there were no substantial reactions before the announcement day since all sectors experienced divergent returns, which is believed to have been exacerbated by information leaks a few days before the announcement. However, the properties sector overreacted, with the stock price plunging by 11.01% following a slight positive abnormal return on the day of the announcement. Most sectors responded positively, with the technology sector yielding a remarkable 24.54% throughout the event window. On the other hand, the financial, energy, and transportation sectors all suffered declines, with the property sector suffering the most, with a decline of 22.01%.

**Table 13:** Results of the impact of travel restrictions during Ramadan on 26 March 2021

Sector name	Sector leader	Abnormal returns			
		Anticipation window (− 3, 0), (%)	Event day (0, 0), (%)	Adjustment window (0, 3), (%)	Event window (− 3, 3), (%)
Financial	BBCA	− 1.33	0.01	− 0.26	− 1.57
Consumer non-cyclical	UNVR	0.85	− 0.78	1.38	1.44
Basic material	TPIA	− 0.39	0.34	4.76	4.72
Infrastructure	TLKM	4.59	0.81	2.35	7.92
Industrial	ASII	0.99	2.33	− 2.26	1.01
Energy	ADRO	− 2.69	0.2	0.48	− 2.02
Consumer cyclical	ACES	− 1.44	0.26	2.07	0.87
Properties and real estate	POLL	− 13.22	0.99	− 11.01	− 22.01*
Healthcare	KLBF	1.76	− 1.71	2.2	2.23
Technology	MCAS	2.95	11.64**	8.36	24.54**
Transportation and logistic	GIAA	− 0.62	− 3.29	2.97	− 1.04

Note: ‘**’ means significance at the 5% level and ‘*’ means significance at the 10% level.

#### Free vaccination campaign

Most sectors expressed support for the free vaccination campaign (Table 14). The reaction on the announcement day was moderate, with an expected significant increase in the healthcare sector’s stock price. Additionally, only the basic material and consumer cyclical sectors showed significant abnormal returns during the anticipation window, at − 6.23% and 7.03%, respectively. Moreover, a signal of underreaction was observed in the technology sector, with the stock price increasing significantly by 29.66% inside the adjustment window. In comparison, we concluded that the considerable reduction in basic material was unrelated to the intervention since the intervention was irrelevant to the sector. Overall, eight out of eleven sectors responded positively to the announcement, with the healthcare and technology sectors bearing the brunt of the impact.

**Table 14:** Results of the impact of free vaccination campaign on 16 December 2020

Sector name	Sector leader	Abnormal returns			
		Anticipation window (− 3, 0), (%)	Event day (0, 0), (%)	Adjustment window (0, 3), (%)	Event window (− 3, 3), (%)
Financial	BBCA	2.17	0.23	− 2.13	0.22
Consumer non-cyclical	UNVR	− 0.69	0.07	2.11	1.47
Basic material	TPIA	− 6.23**	− 1.47	− 5.55**	− 12.74**
Infrastructure	TLKM	4.09	1.66	− 3.22	2.41%
Industrial	ASII	0.31%	0.6%	− 0.76%	0.15%
Energy	ADRO	− 3.3%	− 0.58%	− 1.8%	− 5.59%
Consumer cyclical	ACES	7.03%**	− 2.08%	1.22%	6.08%
Properties & real estate	POLL	11.63%	− 0.61%	− 2.35%	8.34%
Healthcare	KLBF	0.79%	3.82%**	0.58%	5.25%
Technology	MCAS	0.17%	4.16%	29.66%**	35.29%**
Transportation and logistic	GIAA	− 7.32%	− 1.95%	− 1.11%	− 10.13%

Note: ‘**’ means significance at the 5% level and ‘*’ means significance at the 10% level

The Indonesian stock market remained unaffected by the free vaccination campaign. The reaction was entirely contradictory for that in the United Kingdom and the United States, where the FTSE100 and Dow Jones both increased by around 5% on the day of the announcement. Additionally, in some cases, both countries have seen a substantial improvement in the stock prices of airlines, hotels, and energy companies, in some cases by more than 40% (Jack 2020).

Furthermore, Rouatbi et al. (2021) indicated that free vaccination campaigns had decreased global stock market volatility. According to the study, a 10% rise in vaccination might result in a 0.245% positive reaction in the stock market. However, adoption in emerging economies was regarded to be a hurdle to gaining investor confidence. A significant divide between emerging and developed countries could hamper economic growth, as The Economist (2021) estimated that emerging countries would not have widespread access to vaccines until 2023. Emerging economies such as Indonesia and India only have fully vaccinated rates of 11.2% and 9.2%, respectively. In contrast, developed economies such as the United Kingdom and the United States have already surpassed the 50% mark. As a result, the vaccination campaign is a critical factor in a country's economic recovery from the uncertainty during the COVID-19 pandemic.

## Summary and conclusions

This paper examined the short-term impact of government interventions on the industrial sectors of the Indonesian stock market during the COVID-19 pandemic, and the analysis focused on the following five types of interventions: economic stimulus packages, job creation law, Jakarta lockdowns, Ramadan travel restrictions, and free vaccination campaign. The results from the proposed event study analysis indicated that the initial economic stimulus package was critical in reviving the stock market following its collapse to a 7-years low in March 2020. It was also observed that the combination of household and corporate support was the most powerful economic stimulus package. In contrast, the enactment of ****the jobs creation law ushered in a new era of hope for the Indonesian bureaucracy. Although the authorisation had a minor influence on several sectors, it was anticipated that the law would benefit the country’s economy in the future. Furthermore, the Jakarta lockdowns had no noticeable impact on any industrial sector in Indonesia. Indonesia was the last major country in the Southeast Asia region to impose lockdown, which indicated that the Indonesian stock market had experienced spillover impact from other countries’ announcements. Additionally, the September and July lockdowns were less severe than the initial one, showing that the COVID-19 situation affected investors’ behaviour, likely resulting in market resistance during the COVID-19 pandemic. Whereas Ramadan travel restrictions had historically resulted in significant negative sentiment in global stock markets, it was not the case in Indonesia and the announcement had no discernible impact on any industrial sector amid the pandemic. Additionally, the stock market was unaffected negatively by the recurrence of Ramadan travel restrictions in 2021. It was also anticipated that a free vaccination campaign would benefit the Indonesian healthcare sector in the short run. Additionally, the announcement was well received by most sectors. However, it was believed that emerging countries’ lack of access to vaccines could impede gaining market trust. As a result, the intervention’s long-term impact is likely to rely on the government’s commitment to distribute vaccines.

In conclusion, the Indonesian government took a relatively conservative strategy by enforcing a series of government interventions to reduce COVID-19 transmission rates while also stabilising stock market volatility. This was reflected in the stock market’s rapid recovery with a monthly return of 6.53% in December 2020, which was a higher return than other major countries in Southeast Asia, including Singapore (1.13%), Malaysia (4,13%), Thailand (2.91%), and Philippines (5.13%) (Yahoo Finance 2021). Presumably the government had gained knowledge from prior disease outbreaks such as SARS and H1N1, in which Indonesia was involved in the fight against the virus. In addition, Indonesia had also experienced two financial crises in the past 25 years, including the 1997 Asian Financial Crisis and the 2008 Global Financial Crisis, which helped the current government take steps to prevent another crisis.

The event study analysis used in the paper limited the focus to immediate and short-term analysis. However, it would be interesting to examine the longer-term impact of the interventions implemented by the government and further extend the market model to include investor behaviour and political factors in quantifying the short-term impact of recurrence events. Furthermore, due to many interventions implemented in a tight timeframe, an analysis of the impact of combined interventions could also help policy makers to gain better understanding on the formation of effective portfolios of interventions in the event of a pandemic.

## Supplementary Information

Below is the link to the electronic supplementary material.

Supplementary file1 (DOCX 79 kb)

## Data Availability

Publicly accessible data used in the research.

## Code availability

Available on request.
