# Supplementary material for: Impact of government interventions on the stock market during COVID-19: a case study in Indonesia
Source: SN Bus Econ. 2022 Aug 17;2(9):136. doi: 10.1007/s43546-022-00312-4 (PMC9381388; doi:10.1007/s43546-022-00312-4)
Supplement: Supplementary file 1 — Supplementary file1 (DOCX 79 kb) [file 43546_2022_312_MOESM1_ESM.docx]

# Appendix

## Appendix A – Literature regarding the stock market reaction to the COVID-19 pandemic

| **Event** | **Scope** | **Methods** | **Literature** | **Finding** |
| --- | --- | --- | --- | --- |
| China confirmed a COVID-19 outbreak | - China - Asia and Europe | - Event study methodology | - He et al. (2020) - Liu et al. (2020) | - Chinese stock markets did not significantly fluctuate when the Chinese government declared the outbreak. However, the investors were found to be underreacted to the event as 15 days later, the stock markets significantly dropped. - China’s mining and transportation industries experienced the worse effect of the pandemic as both are dependent on each other and struggled with the sudden restrictions. On the other hand, the manufacturing and technology industries were immune to the pandemic due to exceptionally high demand in health-related production and rapid transformation to digital business. - Other Asian indices experienced significant abnormal negative returns as many Asian countries rely heavily on China during the same period. On the other hand, European indices still showed positive returns because the outbreak had not reached Europe on the day of the announcement. This indicated that stock performances negatively correlated to the number of COVID-19 cases. |
| WHO declared COVID-19 as a global pandemic | - Asia - United States - Airline industry | - GARCH (Generalized AutoRegressive Conditional Heteroskedasticity) - Event study methodology | - AlAli (2020) - Harjoto, Rossi, and Paglia (2020) - Maneenop and Kotcharin (2020) | - The WHO announcement was statistically significant to the five of Asia’s largest stock indices. However, investors underreacted on the announcement day, which led to a significant decrease at least 30 days later. - There is an exciting finding in the United States. The WHO announcement severely damaged SMEs with an average of -17.81% within 11 days around the announcement, while large firms still survive a positive return of 0.65% in the same period. The study believed that insufficient support from the US monetary policies was one of the factors that affected the SME’s performance. - The global airline stocks plunged when the WHO declared COVID-19 a global pandemic, as investors panicked and overreacted with the announcement. This resulted in a global accumulative negative return of 24.42% within 11 days around the announcement. - While Europe and US-based airlines were significantly impacted for the period of the announcement, Asia based airlines, like in China and Korea, were less impacted due to several government incentives, including state-backed loans and capital injection to help with debt payments. |
| Countries declared their first COVID-19 cases | - United States - Western Europe | - GARCH - VAR (Vector Autoregressive) - ESM (Event study Method) - Event study methodology | - Chowdhury and Abedin (2020) - Khatatbeh, Hani and Abu-Alfoul (2020) | - The first confirmed cases in the United States created an unstable condition for the short-term until 26 March 2020, in both Dow Jones and S&P500 index. New cases negatively impacted both indexes and a significant increase in the trading volume, which implied many investors withdraw their money. In addition, although S&P500 and Dow Jones were heading in the same direction, S&P500 was inclined to be more volatile, which was shown by a 10% greater loss within 28 days after the first confirmed cases. - Stock markets in Western Europe experienced a short-term shock after the first confirmed cases. However, most have shown a delayed response to the event. For example, the UK held a positive return five days after the first confirmed cases but significantly negative on the 10th day. However, Italy and Spain showed a positive return even after the 10^th^ day. This situation indicated two possible reasons: market efficiency difference and investor confidence in the authorities. |

## Appendix B – Literature regarding the impact on government interventions on the COVID-19 new cases

| **Intervention** | **Scope** | **Methods** | **Literature** | **Impact on COVID-19 cases** |
| --- | --- | --- | --- | --- |
| Contact tracing | - United Kingdom - New Zealand - South Korea - Vietnam | - Agent-based model - Branching process model | - Almagor and Picascia (2020) - Lewis (2020) - James et al. (2021) | - A synergy between contact tracing, testing, and self-isolation policies resulted in a more significant reduction in the transmission rate if the test results can be returned within 24 hours. - Most studies agreed that contact tracing above six days had little benefit. Hence, it should only cover less short time and only traced close contacts and households. - Manual contact tracing can be time-consuming and inefficient despite a well-established system like New Zealand. At the same time, digital tracing apps would be costly and challenging to implement in emerging countries with low adoption of smartphones. - Trust deficit was a significant issue for western countries to access personal data for contact tracing. In contrast, Asian countries like South Korea and Vietnam could access credit cards and mobile phones for emergency tracing. |
| Border restriction | - New Zealand - Australia - Africa | - Stochastic meta-population model - Branching process model - Agent-based model | - Adekunle et al. (2020) - Steyn et al. (2021) - Emeto, Alele, and Ilesanmi (2021) | - Border restriction was highly effective to reduce the number of import cases. However, all studies agreed that it should depend on the country’s underlying condition and targeted travellers from moderate-restrictions countries with a high number of active cases. - For isolated countries like Australia and New Zealand, border restriction was vital to delay and control the COVID-19 pandemic at any stage of the outbreak. Both studies show an 80% reduction in imported cases, allowing the government to focus more on community transmission. Also, 14-day quarantine was deemed to be the best option to avoid the virus entering the communities. - The adoption of border restriction in African countries had a reverse effect to the expected outcome, with nine neighbouring countries recorded an increase in infection rate after the announcement. In addition, many families rely on informal cross-border trade, resulting in high unemployment rates, which led to a negative effect on household welfare and health. |
| School closure | - Global - China - Western Europe | - Regression coefficient - Hierarchical Bayesian Transmission Model | - Jüni et al. (2020) - Viner et al. (2020) - Sharma et al. (2021) | - School closure was found to be effective at the beginning of the COVID-19 pandemic in a study of 144 countries, with a similar reduction rate to social distancing and gathering restrictions. - School closure was likely to control COVID-19 in China, as children tend to suffer asymptomatic cases. However, the actual economic cost and potential harms to the future were undoubtedly high. - There was a significant contribution from school closure in Western European countries during the first wave. However, its effect was insignificant in the second wave, with only 7% compared to social distancing with 35%. |
| Lockdown | - Global | - Regression models | - Koh, Naing, and Wong (2020) - Goldstein, Yeyati, and Sartorio (2021) | - The lockdown-type measures had the most significant effect on limiting the transmission rate in a study of 142 countries before the 100^th^ case was identified. Although an influential factor, the effectiveness of lockdowns tends to decline after 120 days implemented, with no significant effect to reduce the transmission rate. Also, there was no significant difference in implementing partial and complete lockdowns at the beginning of the pandemic. - Emerging countries should impose a brief but strict lockdown to control the transmission rate. Blind lockdown to the national population was also deemed ineffective, as it triggered depression in emerging countries’ economies with many underprivileged populations at risk. The study concluded that data should identify hospital occupancy and another important measure before imposing lockdown. |
| Social distancing | - Global - Europe | - Differential Equations Lead to Predictions of Hospitalisations and Infections (DELPHI) model - Linear-mixed effect models | - Li et al. (2020) - Woskie et al. (2021) | - Stay at home was the most effective policy after being simulated in over 167 countries, with a 25% reduction in transmission rate. If the governments could sustain the social and humanitarian cost, social distancing could suppress the case reproductive number to below one by maintaining the restrictions during the pandemic. - European countries introduced mandatory and non-mandatory social distancing policies at the beginning of the pandemic. However, the model estimated that the outcome of non-mandatory policies was highly dependent on the country’s health system, as low confidence in the health system led to higher adherence to social distancing policies. Hence, the study concluded that countries should impose a mandatory social distancing policy. |
| Vaccination policies | - United Kingdom - Israel | - Mathematical modelling study | - Moore et al. (2021) - Haas et al. (2021) | - In a study in the UK, assuming all adults have received full dose and its efficacy is 85%, vaccination alone is not enough to stop the pandemic, with only 60% effectiveness to reduce the transmission rate. Furthermore, the model predicted a possibility of 24,000 deaths if the government suddenly release non-pharmaceutical restrictions. Hence, the study suggested that gradual release of restrictions is required while waiting for the immune to develop for most people to create herd immunity. - Israel had fully vaccinated over 60% of its population by May 2021. The vaccines offered hope to control the pandemic. The gradual release of lockdown from January 2021 to March 2021 showed a drastic decrease in the 7-day moving average cases to below ten instances per 100,000 population (in all age stages). Additionally, the study suggested that the country’s geographical location played a vital role in the vaccine’s efficacy as the BNT162b2 vaccines successfully took care of asymptomatic and new variants of COVID-19 in Israel. In contrast, other countries might not experience the same. |

## Appendix C – Literature regarding the impact of government interventions on the stock markets

| **Intervention** | **Scope** | **Methods** | **Literature** | **Impact on stock markets** |
| --- | --- | --- | --- | --- |
| Lockdown | - OECD countries - BRICS countries - India | - DSDM (Dynamic Spatial Durbin Model) - Event study | - Eleftheriou and Patsoulis (2020) - Scherf, Matschke and Rieger (2022) - Alam, Alam, and Chavali (2020) | - Both studies agreed that the implementation of lockdowns across the globe had resulted in continuous negative returns to the specific country’s stock market. Additionally, there was a slight spillover effect to the interrelated countries in the short term. For example, when one country in the regions enforced lockdown, most neighbouring countries also followed a slight downturn in the stock market performance. - Markets in both emerging and developed economies were overreacted when the government announced the lockdown with a significant drop in returns but bounced back after a couple of days. Instead, when the government loosened the lockdown, markets were underreacted with no significant impact on the day but subsequently corrected in a few days after the announcement. - India, as one of the largest emerging economies, showed a reverse impact on the global findings. After the announcement, the stock market showed a positive signal with continuous positive returns within two days. However, many believed that markets were cautious before the announcement as the Indian government was late to impose any restrictions. Therefore, the study believed that timing was essential in any restriction’s announcement. |
| Gathering restrictions | - Global - OECD countries - Emerging countries | - Asset pricing model - Panel regression model - Exponential GARCH model | - Zaremba et al. (2020) - Yang and Deng (2021) - Haroon and Rizvi (2020) | - Most of the studies agreed that gathering restrictions caused high volatility in the global stock markets. For example, a slight increase in the stringency measure would cause impactful volatility that ranges from 0.87% to 1.1% in the stock market. Moreover, the cancellation of public events was believed to have the most impactful negative sentiments in the markets as many businesses directly lose their money from this restriction. - The majority of emerging countries in America, Europe, and the Middle East experienced the same effect from the gathering restrictions, which significantly reduced the illiquidity situation. However, the Asian emerging countries showed the opposite reaction with no sign of impacting the market’s liquidity. Thus, the study concluded that Asian markets tend to be structurally different regarding information transparency and liquidity transmission. |
| Economic support | - Global - Europe - Asia | - Panel OLS regression model - Event study | - Ashraf (2020) - Klose and Tillmann (2021) - Beirne et al. (2021) | - The economic support interventions like income support and debt relief resulted in positive market returns in 77 countries across the globe. However, the returns were not statistically significant, which was assumed because the support was directed to households and not directly to businesses. - Monetary policies in 29 European countries were effective in easing the pressure on stock markets. More interestingly, the first announcement did not attract investors, which resulted in stocks fall. Instead, subsequent announcements were getting positive signals from the markets. In contrast, fiscal policies like fiscal stimulus and liquidity packages tend to negatively affect the stock market, while tax-deferrals received reverse reactions. Thus, overall, monetary policies were believed to be more effective to stimulate the stock markets in Europe. - Monetary policies like Quantitative easing and fiscal stimulus packages were also found to be vital to helped boost stock prices in developed and emerging Asian countries. Nevertheless, more interestingly, emerging Asian countries experienced a spillover effect from quantitative easing from developed countries’ central banks with a contribution of 8% at the beginning of the pandemic. |

## Appendix D – The use of event study methodology in various literature

| **Year** | **Authors** | **Topic** | **Nature of Study** | **Scope** | **Methodology** | **Data Frequency** | **Estimation Period** | **Event**  **Window** |
| --- | --- | --- | --- | --- | --- | --- | --- | --- |
| 1969 | Fama, Fisher, Jensen, and Roll | Stock Split | Examine the effect of the announcement of a stock split. | CRSP NYSE Market Portfolio | • FFJR Model | Monthly | 29 months | -6,0,6 |
| 1995 | Agrawal and Kamakura | Corporate Marketing Action | Explore the impact of celebrity endorsers on the companies’ stock. | Sample of 100 events of celebrity endorsements in the US between 1980 to 1992. | • Market Model | Daily | 239 days | -10,0,+10 |
| 2007 | Chen, Jang and Kim | SARS | Examine the effect of SARS on the Taiwanese hotel stock price. | Seven listed hotel companies in the Taiwanese Stock Exchange | • Market Model | Daily | 232 days | -10,0,+10 |
| 2007 | Nikolaos et al. | Merger and Acquisitions | Measure abnormal returns of mergers based on the announcement date. | 6 Greek Industrial and Construction Firms | • OLS Market Model • E-GARCH | Daily | 200 days | -10,0,+10 |
| 2010 | Wai, Kong, Cheung | Corporate Sustainability | Analyse the impacts of the index on sustainable corporate firms in the US stock market. | Sample of 139 firms across eight industries in the US stock market. | • Market Model | Daily | 234 days | Various windows from -15 to +60 days |
| 2014 | Odendaal | Corporate Financial Action | Indicate financial market efficiency when earning announcements. | FTSE100 companies | • CAPM Model | Daily | 120 days | -20,0,+20 |
| 2016 | Cayon, Sarmiento-Sabogal and Shukla | Global Financial Crisis | Measure the effects of news during the global financial crisis (GFC) on Colombia local currency. | High-frequency data on peso-denominated Colombian government bonds | • Market-adjusted Model | Intraday | Six years | Various windows depending on the event |
| 2019 | Bash and Alsaifi | Political Issue | Measure the effect of the uncertain event of Jamal Khashoggi’s disappearance on the Saudi Arabia Stock Exchange. | 157 stocks listed in the Saudi Arabia Stock Exchange | • Market Model • Market-adjusted Model | Daily | 239 days | Various small windows from -10 to +10 days |
| 2020 | Buigut and Kapar | Geopolitical Issue | Examine the impact on geopolitical issues in GCC countries when Qatar was banned in 2017. | Composite index from 7 stock markets in GCC countries | • Market Model | Daily | 250 days | -3,0,+3 0,+3 0,+13 0,+30 |
| 2020 | Tahir et al. | Terrorism | Examine the stock market’s response to terrorist attacks in Pakistan from 2014 - 2017. | Composite index (KSE-100) of Karachi Stock Exchange | • Market Model | Daily | 252 days | -5,0,+10 |
| 2020 | He et al. | COVID-19 | Examine the impact of the COVID-19 outbreak in China on all industrial sectors. | Chinese Stock Market (2,895 companies) | • Market Model | Daily | 160 days | Various small windows from -30 to +30 days |
| 2020 | Maneenop and Kotcharin | COVID-19 | Examine the short-term impact of COVID-19 events in the aviation industry. | 52 Global Listed Airline Companies | • Market Model | Daily | 253 days | -5,0,+5 |
| 2020 | Alam, Wei and Wahid | COVID-19 | Understand the short-term impact of COVID-19 on the Australian Stock Exchange. | Eight industrial sectors in Australia Stock Exchange | • Market-adjusted Model | Daily | 120 days | -10,0,+10 |

## Appendix E – The parametric test results for the event study analysis

(1) The parametric test result for the announcement of the economic stimulus package on 23 March 2020.

| **Sector Name** | **Sector Leader** | **t-statistics Anticipation Window (-3,0)** | **t-statistics Event Day (0,0)** | **t-statistics Adjustment Window (0,3)** | **t-statistics Event Window (-3,3)** | **p-value Anticipation Window (-3,0)** | **p-value Event Day (0,0)** | **p-value Adjustment Window (0,3)** | **p-value Event Window (-3,3)** |
| --- | --- | --- | --- | --- | --- | --- | --- | --- | --- |
| Financial | BBCA | 0.255 | 3.922 | -0.290 | 1.457 | 80.00% | 0.03% | 77.34% | 15.30% |
| Consumer non-cyclical | UNVR | 0.911 | 3.225 | 0.480 | 2.232 | 36.78% | 0.25% | 63.42% | 3.13% |
| Basic material | TPIA | 1.066 | -2.147 | -2.417 | -1.726 | 29.28% | 3.79% | 2.03% | 9.21% |
| Infrastructure | TLKM | 1.398 | 1.281 | 0.042 | 1.451 | 16.98% | 20.76% | 96.63% | 15.46% |
| Industrial | ASII | -2.541 | -1.579 | 0.493 | -1.921 | 1.50% | 12.22% | 62.49% | 6.19% |
| Energy | ADRO | 6.001 | -1.176 | -0.780 | 2.615 | 0.00% | 24.65% | 43.99% | 1.25% |
| Consumer cyclical | ACES | -0.032 | 2.676 | -0.284 | 0.788 | 97.47% | 1.08% | 77.81% | 43.52% |
| Properties & real estate | POLL | -0.112 | -1.612 | 3.080 | 1.225 | 91.13% | 11.48% | 0.37% | 22.77% |
| Healthcare | KLBF | -1.307 | 0.070 | 4.110 | 1.678 | 19.87% | 94.49% | 0.02% | 10.12% |
| Technology | MCAS | -2.374 | -0.475 | -1.941 | -2.798 | 2.25% | 63.70% | 5.93% | 0.79% |
| Transportation and logistic | GIAA | -0.737 | -0.229 | 0.519 | -0.241 | 46.53% | 82.03% | 60.66% | 81.12% |

(2) The parametric test result for the announcement of Jakarta lockdown on 8 April 2020.

| **Sector Name** | **Sector Leader** | **t-statistics Anticipation Window (-3,0)** | **t-statistics Event Day (0,0)** | **t-statistics Adjustment Window (0,3)** | **t-statistics Event Window (-3,3)** | **p-value Anticipation Window (-3,0)** | **p-value Event Day (0,0)** | **p-value Adjustment Window (0,3)** | **p-value Event Window (-3,3)** |
| --- | --- | --- | --- | --- | --- | --- | --- | --- | --- |
| Financial | BBCA | -0.953 | 1.769 | -1.703 | -1.091 | 34.62% | 8.45% | 9.63% | 28.19% |
| Consumer non-cyclical | UNVR | -1.772 | 0.575 | -0.840 | -1.472 | 8.40% | 56.87% | 40.58% | 14.88% |
| Basic material | TPIA | 3.115 | 0.649 | 0.582 | 2.837 | 0.34% | 52.01% | 56.37% | 0.71% |
| Infrastructure | TLKM | -1.281 | -0.022 | 0.343 | -0.632 | 20.77% | 98.27% | 73.37% | 53.13% |
| Industrial | ASII | -0.757 | -0.273 | 0.282 | -0.418 | 45.34% | 78.66% | 77.97% | 67.80% |
| Energy | ADRO | -0.063 | -0.658 | -0.441 | -0.569 | 94.97% | 51.44% | 66.19% | 57.26% |
| Consumer cyclical | ACES | 0.487 | -1.428 | -0.649 | -0.651 | 62.89% | 16.10% | 52.02% | 51.89% |
| Properties & real estate | POLL | -1.347 | -1.211 | -2.091 | -2.478 | 18.56% | 23.31% | 4.30% | 1.75% |
| Healthcare | KLBF | -1.891 | -1.073 | 0.042 | -1.574 | 6.59% | 28.99% | 96.67% | 12.34% |
| Technology | MCAS | 0.103 | 0.085 | 1.366 | 1.002 | 91.85% | 93.29% | 17.95% | 32.24% |
| Transportation and logistic | GIAA | 3.038 | -0.459 | 0.702 | 2.308 | 0.42% | 64.85% | 48.64% | 2.62% |

(3) The parametric test result for the announcement of national travel restriction on 21 April 2020.

| **Sector Name** | **Sector Leader** | **t-statistics Anticipation Window (-3,0)** | **t-statistics Event Day (0,0)** | **t-statistics Adjustment Window (0,3)** | **t-statistics Event Window (-3,3)** | **p-value Anticipation Window (-3,0)** | **p-value Event Day (0,0)** | **p-value Adjustment Window (0,3)** | **p-value Event Window (-3,3)** |
| --- | --- | --- | --- | --- | --- | --- | --- | --- | --- |
| Financial | BBCA | -0.518 | -0.175 | -1.620 | -1.445 | 60.71% | 86.22% | 11.30% | 15.62% |
| Consumer non-cyclical | UNVR | -0.043 | 0.132 | 1.289 | 0.867 | 96.59% | 89.53% | 20.47% | 39.10% |
| Basic material | TPIA | 0.408 | 0.353 | 1.176 | 1.214 | 68.57% | 72.61% | 24.64% | 23.19% |
| Infrastructure | TLKM | 0.280 | 0.010 | -0.009 | 0.182 | 78.07% | 99.18% | 99.30% | 85.68% |
| Industrial | ASII | -0.078 | 0.088 | 0.157 | 0.085 | 93.80% | 93.06% | 87.58% | 93.30% |
| Energy | ADRO | -0.706 | -0.883 | -0.976 | -1.365 | 48.40% | 38.26% | 33.51% | 17.98% |
| Consumer cyclical | ACES | 0.805 | -0.272 | 1.786 | 1.625 | 42.56% | 78.67% | 8.18% | 11.20% |
| Properties & real estate | POLL | 0.439 | 0.335 | -1.430 | -0.565 | 66.30% | 73.95% | 16.05% | 57.49% |
| Healthcare | KLBF | -0.127 | 0.641 | 0.432 | 0.444 | 89.97% | 52.53% | 66.83% | 65.94% |
| Technology | MCAS | -0.029 | 0.223 | 0.842 | 0.619 | 97.69% | 82.49% | 40.46% | 53.92% |
| Transportation and logistic | GIAA | -0.025 | -0.888 | -1.563 | -1.349 | 98.05% | 37.97% | 12.60% | 18.48% |

(4) The parametric test result for the announcement of Jakarta lockdown on 10 September 2020.

| **Sector Name** | **Sector Leader** | **t-statistics Anticipation Window (-3,0)** | **t-statistics Event Day (0,0)** | **t-statistics Adjustment Window (0,3)** | **t-statistics Event Window (-3,3)** | **p-value Anticipation Window (-3,0)** | **p-value Event Day (0,0)** | **p-value Adjustment Window (0,3)** | **p-value Event Window (-3,3)** |
| --- | --- | --- | --- | --- | --- | --- | --- | --- | --- |
| Financial | BBCA | -0.198 | -1.245 | -1.909 | -1.825 | 84.42% | 22.03% | 6.34% | 7.55% |
| Consumer non-cyclical | UNVR | -0.817 | -1.757 | 0.005 | -1.185 | 41.87% | 8.66% | 99.62% | 24.28% |
| Basic material | TPIA | -0.093 | -2.284 | 1.765 | 0.181 | 92.61% | 2.78% | 8.51% | 85.72% |
| Infrastructure | TLKM | 0.355 | 1.668 | 0.771 | 1.385 | 72.42% | 10.30% | 44.55% | 17.37% |
| Industrial | ASII | 0.134 | 1.069 | -0.503 | 0.157 | 89.39% | 29.15% | 61.77% | 87.60% |
| Energy | ADRO | 0.499 | 0.563 | -0.304 | 0.338 | 62.07% | 57.62% | 76.28% | 73.70% |
| Consumer cyclical | ACES | 0.153 | -0.807 | 0.272 | -0.030 | 87.90% | 42.43% | 78.69% | 97.62% |
| Properties & real estate | POLL | 4.387 | 0.422 | 5.646 | 8.952 | 0.01% | 67.52% | 0.00% | 0.00% |
| Healthcare | KLBF | -0.421 | -0.823 | 0.422 | -0.314 | 67.59% | 41.56% | 67.56% | 75.53% |
| Technology | MCAS | -1.586 | -0.498 | 3.344 | 0.719 | 12.07% | 62.14% | 0.18% | 47.66% |
| Transportation and logistic | GIAA | 0.469 | 0.001 | -0.700 | -0.158 | 64.13% | 99.89% | 48.79% | 87.49% |

(5) The parametric test result for the announcement of the authorisation of jobs creation law on 5 November 2020.

| **Sector Name** | **Sector Leader** | **t-statistics Anticipation Window (-3,0)** | **t-statistics Event Day (0,0)** | **t-statistics Adjustment Window (0,3)** | **t-statistics Event Window (-3,3)** | **p-value Anticipation Window (-3,0)** | **p-value Event Day (0,0)** | **p-value Adjustment Window (0,3)** | **p-value Event Window (-3,3)** |
| --- | --- | --- | --- | --- | --- | --- | --- | --- | --- |
| Financial | BBCA | 0.815 | 1.791 | 0.454 | 1.528 | 42.00% | 8.08% | 65.25% | 13.44% |
| Consumer non-cyclical | UNVR | 0.050 | 0.157 | -1.108 | -0.636 | 96.07% | 87.60% | 27.45% | 52.86% |
| Basic material | TPIA | -0.719 | -0.536 | 0.223 | -0.527 | 47.62% | 59.52% | 82.48% | 60.08% |
| Infrastructure | TLKM | -0.186 | 2.473 | -0.176 | 0.688 | 85.32% | 1.78% | 86.10% | 49.54% |
| Industrial | ASII | -0.147 | -1.461 | -0.462 | -0.939 | 88.40% | 15.18% | 64.64% | 35.33% |
| Energy | ADRO | -0.283 | -0.734 | -1.461 | -1.391 | 77.84% | 46.72% | 15.18% | 17.18% |
| Consumer cyclical | ACES | 0.282 | -1.412 | -0.298 | -0.546 | 77.97% | 16.57% | 76.71% | 58.81% |
| Properties & real estate | POLL | -1.355 | -0.097 | -1.292 | -1.577 | 18.31% | 92.35% | 20.37% | 12.27% |
| Healthcare | KLBF | -1.451 | -0.297 | -1.667 | -2.099 | 15.47% | 76.80% | 10.33% | 4.22% |
| Technology | MCAS | -0.413 | -1.210 | -0.333 | -0.915 | 68.19% | 23.35% | 74.10% | 36.56% |
| Transportation and logistic | GIAA | -0.025 | -0.647 | 4.238 | 2.463 | 97.99% | 52.12% | 0.01% | 1.82% |

(6) The parametric test result for the announcement of free vaccination campaign on 16 December 2020.

| **Sector Name** | **Sector Leader** | **t-statistics Anticipation Window (-3,0)** | **t-statistics Event Day (0,0)** | **t-statistics Adjustment Window (0,3)** | **t-statistics Event Window (-3,3)** | **p-value Anticipation Window (-3,0)** | **p-value Event Day (0,0)** | **p-value Adjustment Window (0,3)** | **p-value Event Window (-3,3)** |
| --- | --- | --- | --- | --- | --- | --- | --- | --- | --- |
| Financial | BBCA | 1.314 | 0.245 | -1.293 | 0.088 | 19.64% | 80.78% | 20.34% | 93.05% |
| Consumer non-cyclical | UNVR | -0.380 | 0.064 | 1.159 | 0.529 | 70.58% | 94.90% | 25.35% | 59.97% |
| Basic material | TPIA | -3.070 | -1.256 | -2.734 | -4.108 | 0.38% | 21.65% | 0.93% | 0.02% |
| Infrastructure | TLKM | 1.203 | 0.846 | -0.947 | 0.465 | 23.60% | 40.24% | 34.93% | 64.48% |
| Industrial | ASII | 0.086 | 0.290 | -0.210 | 0.028 | 93.18% | 77.32% | 83.49% | 97.79% |
| Energy | ADRO | -0.827 | -0.253 | -0.450 | -0.917 | 41.34% | 80.17% | 65.50% | 36.47% |
| Consumer cyclical | ACES | 2.205 | -1.132 | 0.383 | 1.249 | 3.32% | 26.43% | 70.35% | 21.90% |
| Properties & real estate | POLL | 0.918 | -0.083 | -0.186 | 0.431 | 36.44% | 93.42% | 85.36% | 66.90% |
| Healthcare | KLBF | 0.360 | 3.023 | 0.267 | 1.570 | 72.07% | 0.43% | 79.11% | 12.43% |
| Technology | MCAS | 0.024 | 1.022 | 4.204 | 3.275 | 98.06% | 31.30% | 0.01% | 0.22% |
| Transportation and logistic | GIAA | -1.004 | -0.464 | -0.152 | -0.910 | 32.15% | 64.50% | 88.00% | 36.82% |

(7) The parametric test result for the announcement of economic stimulus package on 4 January 2021.

| **Sector Name** | **Sector Leader** | **t-statistics Anticipation Window (-3,0)** | **t-statistics Event Day (0,0)** | **t-statistics Adjustment Window (0,3)** | **t-statistics Event Window (-3,3)** | **p-value Anticipation Window (-3,0)** | **p-value Event Day (0,0)** | **p-value Adjustment Window (0,3)** | **p-value Event Window (-3,3)** |
| --- | --- | --- | --- | --- | --- | --- | --- | --- | --- |
| Financial | BBCA | 0.807 | -1.310 | 0.664 | 0.460 | 42.46% | 19.76% | 51.05% | 64.78% |
| Consumer non-cyclical | UNVR | 0.049 | 1.118 | -1.720 | -0.685 | 96.12% | 27.03% | 9.32% | 49.72% |
| Basic material | TPIA | -0.747 | 1.512 | 2.078 | 1.437 | 45.95% | 13.83% | 4.41% | 15.86% |
| Infrastructure | TLKM | 0.341 | 0.893 | -1.119 | -0.190 | 73.50% | 37.72% | 26.96% | 85.05% |
| Industrial | ASII | 0.391 | 0.434 | -0.922 | -0.195 | 69.79% | 66.68% | 36.21% | 84.66% |
| Energy | ADRO | -0.901 | -0.444 | -1.164 | -1.480 | 37.30% | 65.95% | 25.14% | 14.66% |
| Consumer cyclical | ACES | -0.766 | -1.505 | 1.229 | -0.296 | 44.81% | 14.02% | 22.64% | 76.86% |
| Properties & real estate | POLL | -0.284 | -0.408 | -0.496 | -0.639 | 77.80% | 68.54% | 62.28% | 52.63% |
| Healthcare | KLBF | 0.929 | -0.705 | 0.520 | 0.680 | 35.84% | 48.47% | 60.62% | 50.04% |
| Technology | MCAS | 0.428 | 1.004 | 0.726 | 1.178 | 67.09% | 32.12% | 47.20% | 24.59% |
| Transportation and logistic | GIAA | -1.284 | -0.748 | -0.728 | -1.513 | 20.65% | 45.88% | 47.07% | 13.81% |

(8) The parametric test result for the announcement of national travel restriction on 26 March 2021.

| **Sector Name** | **Sector Leader** | **t-statistics Anticipation Window (-3,0)** | **t-statistics Event Day (0,0)** | **t-statistics Adjustment Window (0,3)** | **t-statistics Event Window (-3,3)** | **p-value Anticipation Window (-3,0)** | **p-value Event Day (0,0)** | **p-value Adjustment Window (0,3)** | **p-value Event Window (-3,3)** |
| --- | --- | --- | --- | --- | --- | --- | --- | --- | --- |
| Financial | BBCA | -0.604 | 0.011 | -0.118 | -0.467 | 54.95% | 99.11% | 90.67% | 64.30% |
| Consumer non-cyclical | UNVR | 0.347 | -0.550 | 0.559 | 0.383 | 73.08% | 58.52% | 57.92% | 70.35% |
| Basic material | TPIA | -0.112 | 0.171 | 1.377 | 0.892 | 91.16% | 86.51% | 17.62% | 37.77% |
| Infrastructure | TLKM | 1.416 | 0.434 | 0.725 | 1.599 | 16.45% | 66.67% | 47.27% | 11.77% |
| Industrial | ASII | 0.363 | 1.479 | -0.828 | 0.242 | 71.82% | 14.71% | 41.24% | 81.01% |
| Energy | ADRO | -0.907 | 0.120 | 0.163 | -0.446 | 36.96% | 90.54% | 87.10% | 65.82% |
| Consumer cyclical | ACES | -0.406 | 0.129 | 0.585 | 0.160 | 68.67% | 89.78% | 56.21% | 87.34% |
| Properties & real estate | POLL | -1.582 | 0.204 | -1.318 | -1.725 | 12.15% | 83.91% | 19.51% | 9.23% |
| Healthcare | KLBF | 0.520 | -0.874 | 0.652 | 0.431 | 60.59% | 38.75% | 51.82% | 66.86% |
| Technology | MCAS | 0.567 | 3.877 | 1.607 | 3.089 | 57.38% | 0.04% | 11.60% | 0.36% |
| Transportation and logistic | GIAA | -0.129 | -1.176 | 0.613 | -0.140 | 89.81% | 24.65% | 54.32% | 88.91% |

(9) The parametric test result for the announcement of Jakarta lockdown on 1 July 2021.

| **Sector Name** | **Sector Leader** | **t-statistics Anticipation Window (-3,0)** | **t-statistics Event Day (0,0)** | **t-statistics Adjustment Window (0,3)** | **t-statistics Event Window (-3,3)** | **p-value Anticipation Window (-3,0)** | **p-value Event Day (0,0)** | **p-value Adjustment Window (0,3)** | **p-value Event Window (-3,3)** |
| --- | --- | --- | --- | --- | --- | --- | --- | --- | --- |
| Financial | BBCA | -1.271 | -0.279 | -0.041 | -0.962 | 21.11% | 78.15% | 96.72% | 34.20% |
| Consumer non-cyclical | UNVR | 0.184 | 3.041 | -1.047 | 0.547 | 85.52% | 0.42% | 30.12% | 58.76% |
| Basic material | TPIA | -0.266 | -0.097 | -0.841 | -0.752 | 79.17% | 92.32% | 40.52% | 45.62% |
| Infrastructure | TLKM | -1.003 | -1.113 | -1.564 | -2.045 | 32.17% | 27.22% | 12.57% | 4.74% |
| Industrial | ASII | 0.526 | 0.962 | -1.069 | -0.007 | 60.18% | 34.17% | 29.16% | 99.46% |
| Energy | ADRO | -1.660 | -0.300 | 1.327 | -0.387 | 10.48% | 76.58% | 19.21% | 70.10% |
| Consumer cyclical | ACES | -1.479 | -0.033 | 5.212 | 2.247 | 14.69% | 97.39% | 0.00% | 3.02% |
| Properties & real estate | POLL | 0.253 | 3.527 | 0.565 | 2.010 | 80.12% | 0.11% | 57.54% | 5.12% |
| Healthcare | KLBF | 1.172 | 0.030 | -0.537 | 0.413 | 24.82% | 97.60% | 59.43% | 68.17% |
| Technology | MCAS | 0.450 | -0.878 | -0.585 | -0.430 | 65.54% | 38.50% | 56.20% | 66.96% |
| Transportation and logistic | GIAA | 0.751 | 0.265 | 0.522 | 0.950 | 45.72% | 79.25% | 60.47% | 34.80% |
